# Radiation combined with ultrasound and microbubbles: A potential novel strategy for cancer treatment

**DOI:** 10.1016/j.zemedi.2023.04.007

**Published:** 2023-08-14

**Authors:** Deepa Sharma, Kai Xuan Leong, Daniel Palhares, Gregory J. Czarnota

**Affiliations:** a Physical Sciences, Sunnybrook Research Institute, Toronto, Ontario, Canada; b Department of Radiation Oncology, Sunnybrook Health Sciences Centre, Toronto, Ontario, Canada; c Departments of Radiation Oncology, and Medical Biophysics, University of Toronto, Toronto, Ontario, Canada

**Keywords:** Tumor vasculature, Radiation, Ultrasound-Stimulated Microbubbles, Radiosensitization, Ceramide, Endothelial cells, Acid sphingomyelinase

## Abstract

Cancer is one of the leading causes of death worldwide. Several emerging technologies are helping to battle cancer. Cancer therapies have been effective at killing cancer cells, but a large portion of patients still die to this disease every year. As such, more aggressive treatments of primary cancers are employed and have been shown to be capable of saving a greater number of lives. Recent research advances the field of cancer therapy by employing the use of physical methods to alter tumor biology. It uses microbubbles to enhance radiation effect by damaging tumor vasculature followed by tumor cell death. The technique can specifically target tumor volumes by conforming ultrasound fields capable of microbubbles stimulation and localizing it to avoid vascular damage in surrounding tissues. Thus, this new application of ultrasound-stimulated microbubbles (USMB) can be utilized as a novel approach to cancer therapy by inducing vascular disruption resulting in tumor cell death. Using USMB alongside radiation has showed to augment the anti-vascular effect of radiation, resulting in enhanced tumor response. Recent work with nanobubbles has shown vascular permeation into intracellular space, extending the use of this new treatment method to potentially further improve the therapeutic effect of the ultrasound-based therapy. The significant enhancement of localized tumor cell kill means that radiation-based treatments can be made more potent with lower doses of radiation. This technique can manifest a greater impact on radiation oncology practice by increasing treatment effectiveness significantly while reducing normal tissue toxicity. This review article summarizes the past and recent advances in USMB enhancement of radiation treatments. The review mainly focuses on preclinical findings but also highlights some clinical findings that use USMB as a therapeutic modality in cancer therapy.

## Radiation-induced vascular effects

At present, radiation is used as one of the common forms of cancer treatment. It has been well established that radiation act primarily by inducing DNA damage, subsequently causing cancer cell death [1], [2], [3]. Some studies, on the other hand, suggest vascular destruction as a prime cause of radiation-induced tumor cell death [4], [5], [6]. Single large doses of radiation have a profound effect in damaging endothelial cells, causing apoptosis, leading to tumor cell death as a secondary effect [5], [6], [7], [8]. In studies conducted by Garcia-Barros *et al*. [6] it was suggested that early microvascular endothelial apoptosis is an important factor for tumor cure. Although these results are controversial, the suggested theory is that the damage caused by ionizing radiation in tumor cells is not lethal in themselves, but their conversion to lethality is connected to endothelial cell function and the tumor vasculature. Their data suggested that exposures to single doses of radiation (>8–10 Gy) result in endothelial cell death in a ceramide-dependent manner. Alternatively, fractionated radiation (1.8–3 Gy/fraction) yields effects primarily through tumor cell DNA damage [9], [10]. Ceramide is a lipid molecule that is associated with the induction of apoptotic signaling cascade and has been shown to play a role in the activation of the endothelial cell death in response to high dose exposures [5], [6], [11], [12].

Recent studies have highlighted the important role of the vasculature on tumor responses to radiation therapy (XRT) [13], [14], [15], [16]. It has been demonstrated that ceramide and its downstream metabolite, sphingosine-1 phosphate (S1P) are important in affecting vascular responses to XRT impacting tumor control [17], [18], [19], [20]. Recent work has showed that the use of ultrasound-stimulated microbubbles (USMB) can synergize with XRT to induce vascular destruction, in a ceramide-S1P dependent manner [21], [22]. This research is consistent with the hypothesis that effective perturbation of endothelial cells leads to microvascular destruction, that can enhance radiation effects [22], [23]. Recent studies have revealed that ultrasound and microbubbles-mediated mechanical perturbation of endothelial cells can amplify the effectiveness of radiation significantly. USMB enhancement of radiation response has been documented in several *in vitro* [24], [25], [26] and *in vivo* studies using varieties of tumor models [21], [27], [28], [29], [30]. This methodology takes the approach of perturbing the endothelial lining of the vasculature with highly effective biophysical ultrasound/microbubbles-mediated perturbations instead of anti-angiogenic or pharmacological agents, which have mostly angiostatic effects with limited clinical success and impact.

In recent work, scientists from Thomas Jefferson University have undertaken pre-clinical and clinical work with trans-arterial radioembolization (TARE) in patients leading to improved cancer care results [31], [32], [33]. Results are reviewed fully elsewhere [34], [35], [36].

## Ultrasound-stimulated microbubbles (USMB)

Microbubbles are gas-filled spheres that range between 0.5-10 µm in size and are usually composed of air or a perfluorocarbon, stabilized by a thin shell of biocompatible material, composed of proteins or lipids [37], [38], [39], [40]. Currently, several agents are approved by the Food and Drug Administration (FDA) for clinical use including Albunex, Definity, Echovist, Imagent, Levovist, Optison, SonoVue, Sonozoid (reviewed in [41]). In this review, most of the studies utilizing Definity microbubbles are discussed. Definity microbubbles (Lantheus Medical, USA), are filled with perfluoropropane stabilized within a lipid shell. It has been primarily utilized as an ultrasound contrast agent in multiple fields including echocardiography in adult and pediatrics, liver ultrasound imaging, abdominal interventions and, more [42], [43], [44], [45], [46], [47]. When exposed to ultrasound of specific frequencies and power, microbubbles respond by rapidly expanding and contracting by the effect of a process known as cavitation. There are two types of cavitation; stable cavitation and inertial cavitation. The state of cavitation is highly dependent on several properties of the ultrasound waves, including; frequency, power, duty cycle, mechanical index, and ultrasound exposure, as well as the microbubbles composition and environmental conditions [48], [49], [50], [51]. In a state of stable cavitation, microbubbles exposed to low-power and mechanical index ultrasound fields undergo a stable oscillating pattern that causes the bubble to expand and compress in response to the pressure applied by the ultrasound field. This can cause pushing and pulling forces on the surrounding environment that can lead to microstreaming; a phenomenon in which the change in microbubble diameter generates forces upon the surrounding fluid environment, leading to non-linear fluid dynamics that can interact with the surrounding environment [52], [53], [54], [55]. Increasing the ultrasound power at a high mechanical index results in microbubbles undergoing a less uniform expansion and compression pattern known as inertial cavitation. This unstable form of cavitation can lead to microbubbles collapse, resulting in secondary phenomena such as the generation of shock waves or microjets into the surrounding environment. Often, these effects can generate great amounts of force to surrounding endothelial cells, causing large perforations in the plasma membrane or widening the gaps between cells [56], [57], [58], [59]. The ability of ultrasound to elicit bioeffects is found to be dependent on the behavior of microbubbles at different acoustic pressures. The selection of ultrasound parameters, duty cycle, and microbubbles concentration seems to play a key role in enhancing the tumor effect. Fan *et al.* conducted an *in vitro* study to explore the behavior and effect of low and high ultrasound pressure on cell viability using human umbilical vein endothelial cells (HUVECs). They found the cells exposed to parameters comprising acoustic pressure (0.06 MPa, duty cycle of 20%, pulse repetition frequency of 20 Hz, and 10 ms) resulted in no microbubbles disruption, causing the cells to remain intact. On the other hand, using higher pressure of 0.43 MPa, 20% duty cycle, 20 Hz pulse repetition frequency, and 1 s total duration caused bubbles coalescing, resulting in greater cell damage [60]. Similarly, He *et al.* conducted an *in vivo* study using VX2 tumor inoculated in rabbits to explore the effect of varying ultrasound peak negative acoustic pressure (1.0, 2.0, 3.0, 4.0, or 5.0 MPa). Their results showed a gradual statistical decrease in the tumor blood flow with an increase in acoustic pressure of 2.0, 3.0, 4.0, and 5.0 MPa. Additionally, greater tumor cell death and vessel damage were reported at a higher pressure of 4.0, and 5.0 MPa. They found the optimal acoustic pressure to be 4.0 MPa, at which a significant anti-tumor effect was observed, with no damage seen around the surrounding tissue [61]. Several other studies have also looked at the effect of different ultrasound pressure [62], [63], [64], [65], [66]. The effect of different microbubble concentrations on tumor vascular response has also been explored. It was found that increasing the microbubble concentrations (from 1% to 3%) with an increase in ultrasound pressure can significantly enhance tumor response by causing vascular collapse, followed by tumor cell death. This effect has been documented in various *in vivo* tumor types [21], [22], [27]. Collectively, these studies strongly suggest that the acoustic cavitation that occurs upon high ultrasound pressure causes microbubbles disruption, enhancing the tumor effect. Another proposed mechanism for greater tumor response following high-intensity focused ultrasound (HIFU) is the mechanical disruption caused to the tumor tissue using repeated short-duration pulses with low-duty cycles [67]. Together, all these abovementioned findings provide strong evidence that the permutations of ultrasound pressure and microbubble concentrations might be an essential factor to consider while treating tumors.

## Non-invasive monitoring of treatment response

The use of USMB therapy is an effective way of inducing vascular damage that leads to tumor cell death. Working *in vitro* (HUVEC) *and in vivo* (general schematic) models of USMB enhancement are presented in Figure 2, Figure 3 [21]. In this mechanism, radiation-induced DNA damages encompass both tumor-cell DNA damage and vessel destruction, rather than tumor cell on its own, and subsequent tumor apoptosis and necrosis [68], [69]. The massive ischemic event triggered by the USMB approach goes beyond a traditional hypoxia-driven mechanism and takes it a step further to suggest that, in fact, it is anoxia that results in cell death. While it is known that hypoxia can attenuate the effects of radiation-induced DNA damage, histological evidence has highlighted that complete cell death as a result of anoxic conditions results in a superior response observed experimentally. Thus, the central rationale behind the approach posits that anoxic conditions leading to cell death do not require therapeutic doses of radiation as massive and complete destruction are already induced, whereas areas with partially functioning vasculature can supply oxygen to tumor cells, making radiation more effective in the oxygen-rich environments [16], [70], [71]. Endothelial cells exposed to USMB and radiation experience microbubbles-induced membrane damage that can enhance radiation responses (related to acid sphingomyelinase (ASMase)-dependent ceramide increases as described in [26] and follow-on publications). This microbubbles-induced membrane damage explains the synergistic physiological effects of USMB and radiation on blood vessel acoustical stimulation, which is not seen in *in vitro* experiments.

**Figure 1:**
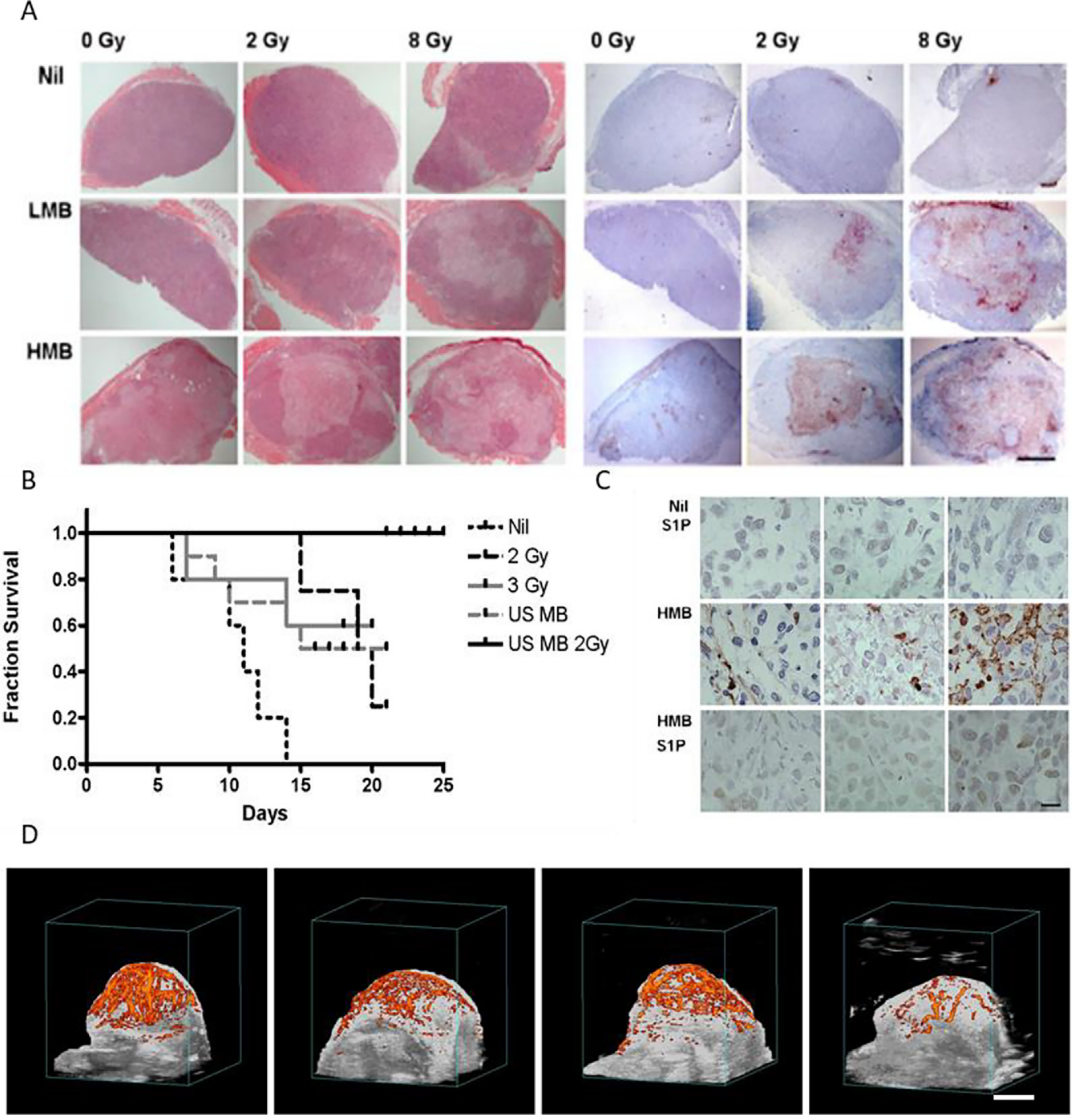
Treatment response evaluation in PC3 tumor xenografts following ultrasound-stimulated microbubbles (USMB) and radiation. (A) Assessments of cell death; left panels depict hematoxylin and eosin (H&E) staining, right panels depict in situ end labeling (ISEL) staining. First row displays nil, no microbubbles, the middle row displays LMB (low microbubble concentration), bottom row displays high microbubble concentration (HMB). The scale bar represents 2 mm. (B) Kaplan-Meier survival curves of mice exposed to multiple fraction treatments. Treatment included 2 Gy fractions (24 Gy in 12 fractions over 3 week) [BED (10) = 28.8], 2 Gy fractions with two USMB treatments weekly, 3 Gy fractions (45 Gy in 15 fractions over 3 week) [BED (10) = 58.5], and USMB treatments weekly (twice weekly for 3 week). (C) Representative higher-magnification images of ISEL–stained PC3 tumor cross-sections. Top row displays tumors treated with sphingosine-1-phosphate (S1P) without the presence of USMB. Middle row displays tumors treated with USMB and radiation in the absence of S1P. Last row displays tumors treated with USMB and radiation in the presence of S1P. Increase in ISEL staining can be seen in the middle row without S1P while presence of S1P diminished the cell death (bottom). The scale bar represents 50 microns. (D) Representative results of power Doppler signal in volumetric images for various treatment conditions assessed at 24 h. Group includes no treatment, microbubbles alone, 8 Gy radiation alone, and USMB and 8 Gy radiation (these are depicted from left to right). The scale bar represents 5 mm. From [21].

**Figure 2:**
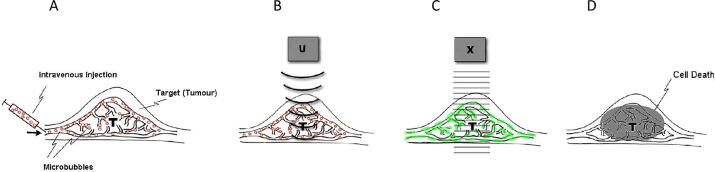
Schematic representation for ultrasound microbubble treatments and its stimulation in radiation enhancement effects. (A) Microbubbles are injected intravenously into the tumor tissue (T) that moves through the vasculature, including capillaries. (B) Ultrasound (U) is applied to initiate cavitation that causes vibration and collapse of gas-filled bubbles into tiny fragments by the ultrasound beam. (C) Bubble cavitation results in endothelial vascular perturbation. (D) Tumors exposed to radiation therapy (X) after USMB treatments result in significant cell death within 24 h after treatment administration. From [21].

**Figure 3:**
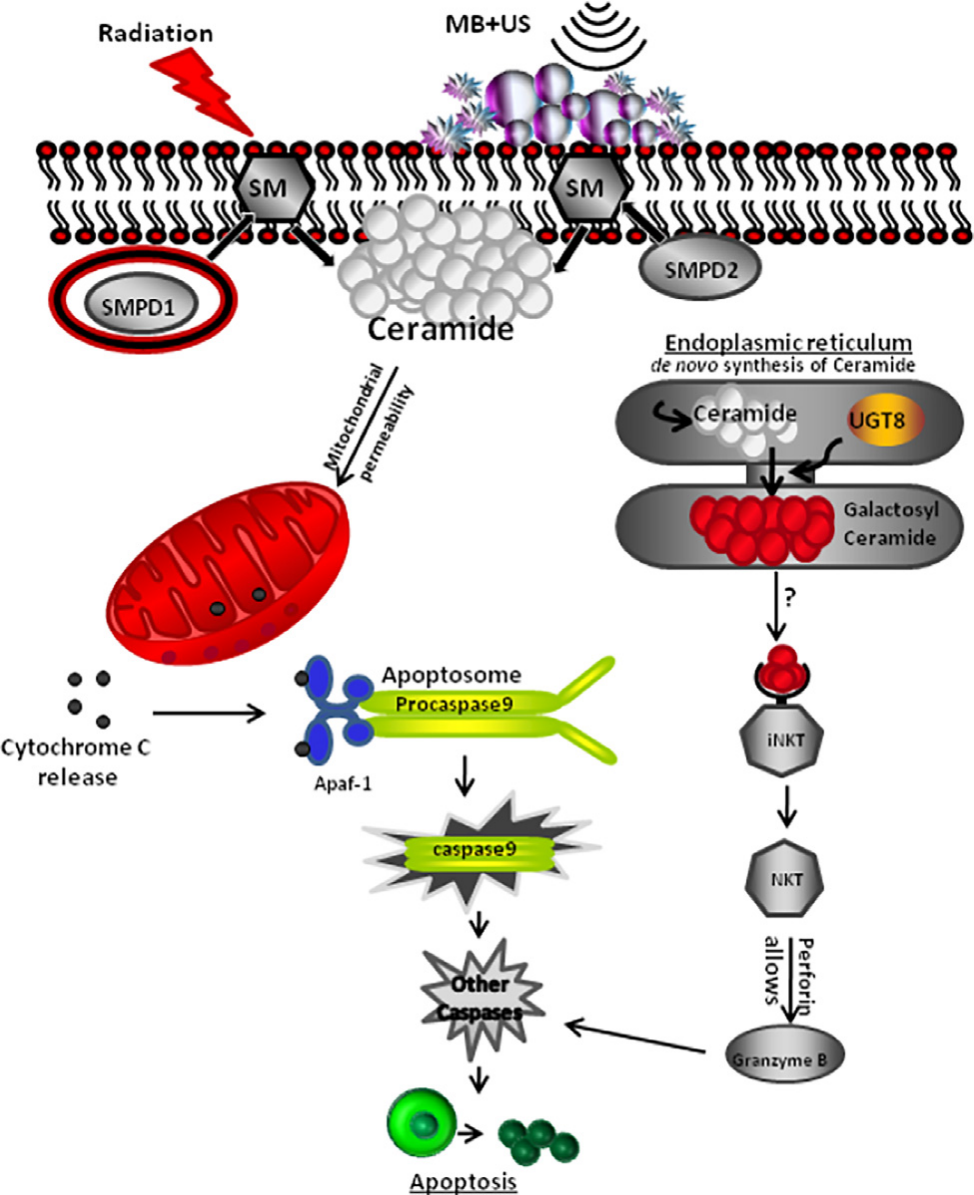
Schematic model depicting apoptotic signaling pathways in HUVEC cells in response to combined USMB radiation treatments. Hydrolysis of sphingomyelin catalyzed by the enzyme sphingomyelinase (sphingomyelin phosphodiesterase 1 (SMPD1) and (sphingomyelin phosphodiesterase 2 (SMPD2)) results in ceramide production. The increased intracellular ceramide stimulates the release of mitochondrial cytochrome c promoting activation of procaspase 9 by inducing nucleotide binding to Apaf-1. Alternatively, De novo-synthesized ceramide is converted to galactosylceramide by UGT8 (UDP Glycosyltransferase 8). Galactosylceramide activates the iNKT cells which secrete perforin that assembles at the cell plasma membrane, allowing granzyme B to cause activation of the apoptotic cascade. From [25].

Tumor selectivity is also achieved with this method, with the ability to focus the ultrasound beam on the tumor volumes while leaving the surrounding tissues unaffected. Modern ultrasound technologies permit acoustic fields to be focused down as small as 1 mm spatial precision and spot size and can be delivered alone or using magnetic resonance imaging (MRI) guidance for clinical treatments, thus avoiding normal tissue toxicity [72], [73], [74]. This allows focused ultrasound (FUS) to be a flexible modality in targeting specific volumes for therapeutic uses. Because of the inert nature of microbubbles when not under the influence of an ultrasound field, areas exposed to microbubbles without ultrasound have negligible cytotoxicity, as observed in several published animal experiments and further supported by the safe use of them in diagnostic imaging [38], [75], [76], [77]. Thus, the ultrasound effects are localized only in the treated area. Experiments using higher power, high-intensity FUS has showed precise tissue heating of 80–90 °C only seen in the focal spot of the ultrasound beam, with a penumbra of micrometers. No effects were seen in intervening tissues [78]. The same principles apply with the lower powers used for USMB treatments.

## USMB enhanced radiation effect

The first *in vivo* experiments involving radiation and microbubbles treatments demonstrated a synergistic physiological effect dependent on blood vessel interaction with acoustically stimulated microbubbles. Cell death was assessed 24 h after treatment that indicated an increase from 5% cell death with 2 Gy of radiation alone to nearly 50% when USMB treatment was administered before 2 Gy radiation exposure ([21], Fig. 1A&B). The pattern of central effect (regions of apoptotic and necrotic cell death) and viable rim in tumors resulting from single treatments was suggestive of an effect similar to that of chemical vascular disrupting agents. Furthermore, multiple treatments consisting of combined USMB and radiation fractions resulted in tumor regression ([21], Fig. 1A&B). Survival experiments (Fig. 1B) suggested that the combination of USMB and radiation was capable of elevating the impact of a non-curative dose of radiation into one with more effect than a curative dose of radiation. Survival of animals bearing PC3 tumors was superior when treated with USMB and 24 Gy of radiation, a non-curative dose of radiation [biological effective dose (BED10 = 28.8 Gy)] as compared to animals exposed to radiation alone, more effective than a curative dose of radiation (BED10 = 58.5 Gy) [21]. In addition, evidence of a viable rim often seen in treatments with chemical anti-angiogenic agents was not observed in these combined USMB and radiation treatments. Instead, vasculature disruption and vascular collapse were observed within the tumor due to endothelial cell death followed by subsequent tumor cell death [21], [27], [30] (reviewed in [34], [35], [79]). Further experiments looking at fractionated XRT combined with USMB in mice and rabbits have expanded upon our initial results, confirming enhanced cell death and superior animal survival when USMB treatments were combined with single radiation treatments or fractionated XRT, magnifying induced cell death (described further below) [80], [81].

The synergistic effect between USMB and radiation has been previously demonstrated to arise from mechanical damage inflicted upon endothelial cells via microbubbles cavitation when stimulated by an ultrasound field [21]. This USMB-induced mechanical damage activates the ASMase-ceramide pathway that plays an important role in facilitating membrane-damage-responses. Membrane perforations generated by USMB result in ASMase-dependent increases in ceramide, which activate endothelial cell death when combined with low (2 Gy) doses of radiation. The radio-enhancing effect conveyed by microbubbles can be inhibited by manipulating the ASMase pathway genetically or chemically and has been observed *in vitro* and *in vivo* in tumor models [21], [22]. This highlights the importance of ASMase in facilitating USMB-based effects. Mechanistic schematics are provided in Figure 2, Figure 3. To monitor alterations in tumor vasculature non-invasively, effects on blood flow were investigated using Doppler ultrasound and other modalities [21], [22]. A recent experimental study conducted with ultrafine bubbles (nanobubbles) combined with ultrasound, indicated greater radio-enhancement effects compared to that with microbubbles [82].

Recent work has scaled up this methodology to be applicable for treatment systems that can be used in clinical settings alongside ultrasound-imaging guidance, and independently with MRI-guidance, for large animal tumor models and first-in-human cancer patient treatments [80], [83], [84].

## Large animal experiments with USMB and XRT

**Figure 4:**
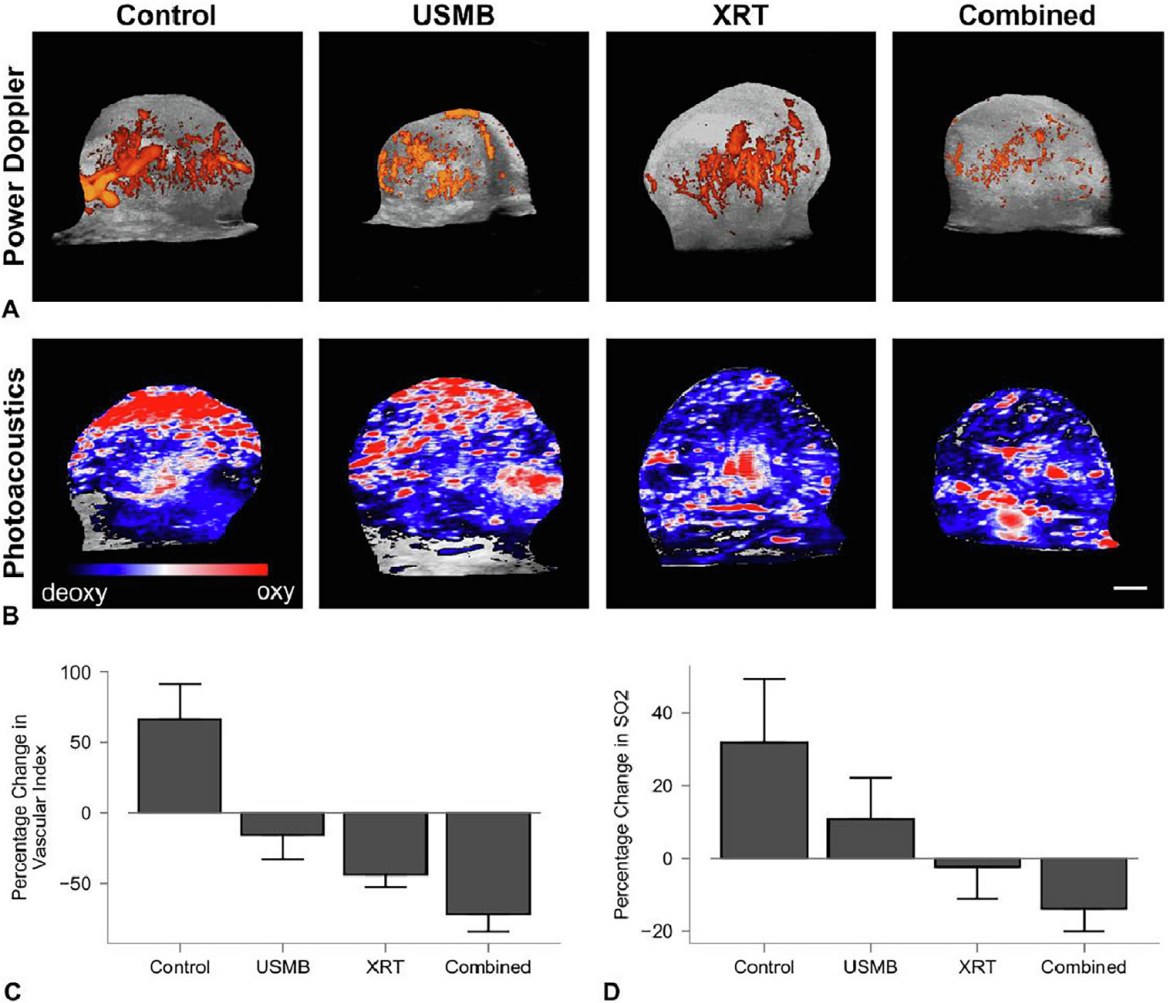
(A) Volumetric 3D power Doppler and (B) photoacoustic post-treatment images of PC3 xenograft tumors *in vivo* 24 h after treatments. For power Doppler imaging, colored overlays indicate 0–40 dB. For photoacoustic imaging, the blue and red color bar represents a relative ratio of oxy- and deoxyhemoglobin levels, respectively. The scale bar represents 4 mm. Treatment conditions included untreated controls, 1% (v/v) microbubble and focused ultrasound exposure (USMB), (XRT, 8 Gy), and combined treatments (USMB + XRT). (C) Percent change in vascular index depicting diminishment in blood flow following combined treatment compared to control groups. (D) Percent change in oxygen saturation showing oxygen saturation reduction in combined treated group comparing control groups. Error bars represent the standard error of the mean (SEM) [80].

**Figure 5:**
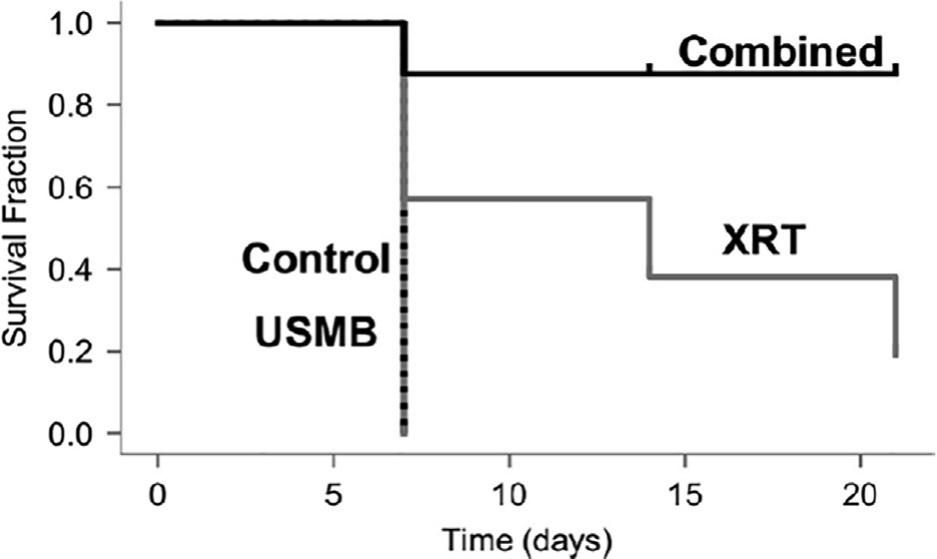
Treatment evaluation for multiple fraction experiments. Kaplan-Meier survival data for multiple fraction treatments, including 1% (v/v) microbubble and focused ultrasound exposure (USMB), multi-fraction radiation (XRT), and combined treatments (USMB + XRT). Animals with untreated tumors or control groups were also included. Animals received USMB treatments twice weekly, and XRT administration in a radiation-only group or combined treatment groups were delivered in five fractions/week at 2 Gy each over 3 weeks (BED10 = 30 Gy). The statistical test revealed a drop of zero in the mean survival after one week for the control group and USMB treatments, whereas 19% diminishment for the XRT group by the end of the third week. A drop of 88% was observed in the combined treatment group after the first week, which persisted for all subsequent weeks. The mean survival changes in the combined group were statistically significant compared to other groups. From [80].

**Figure 6:**
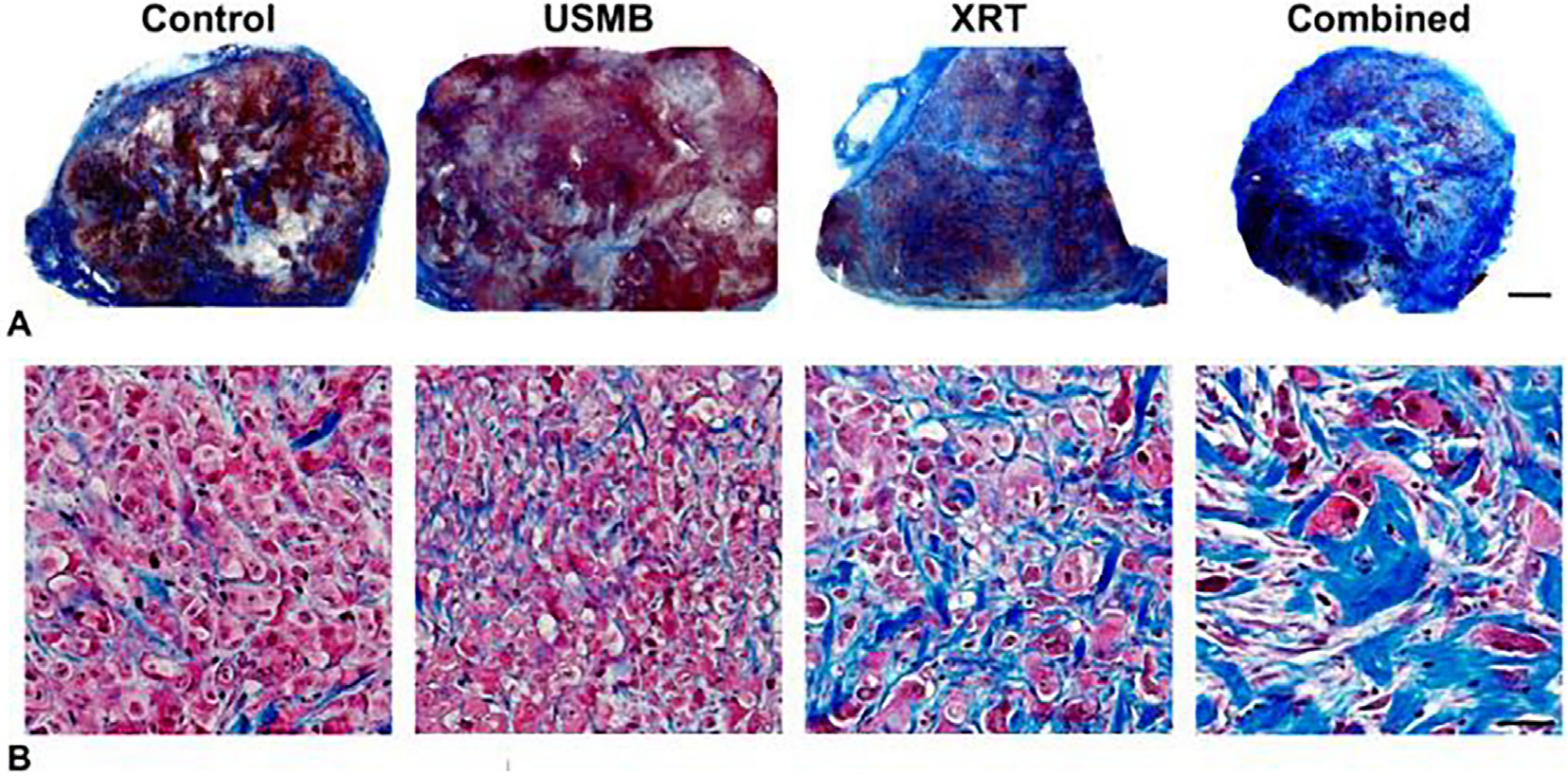
Fibrosis: (A) and (B) Representative low and high magnification images of Masson trichrome-stained tumor cross-sections of PC3 xenograft, respectively. Treatments condition included ultrasound-stimulated microbubbles (USMB; 1%), multi-fraction radiation (XRT), and combined (USMB + XRT) treatments. Animals were administered with USMB treatments twice weekly, and for XRT only and combined treatments, animals were exposed to five fractions/week at 2 Gy each over 3 weeks (BED10 = 30 Gy). Increased fibrotic tissues were observed by the third week in tumors receiving combined treatment. The scale bar represents 2 mm for low-magnification images and 50 μm for high-magnification images. From [80].

Though previous works have primarily utilized smaller rodent models for experiments, recent work has expanded experiments to larger tumor models in rabbits [80]. New Zealand white rabbits bearing prostate tumor (PC3) xenografts were treated with USMB alone, ionizing radiation (XRT; 8 Gy), or a combination of both treatments (USMB + XRT). Treatment responses were assessed at 24 h utilizing immunohistopathology, 3D- power Doppler ultrasound, and photoacoustic imaging. A second cohort of rabbits was treated with a fractionated radiation treatment regimen of 2 Gy doses delivered daily over three weeks. A subset of these rabbits underwent USMB treatments that were delivered twice weekly alongside daily XRT treatments. Tumors receiving combined treatments showed a significant decrease in vascular structure, as indicated by the lack of CD31 staining and visible vascular architecture compared to control and single treatment groups. In conjunction with this, there was an increase in cell death (in situ end-labeling (ISEL)), a decrease in a vascular index (power Doppler imaging), and oxygen saturation (photoacoustic imaging). For long-term fractionated combined treatment in rabbits, there was a significant growth delay after one week, and a significant tumor size reduction after three weeks with the combined treatments (Figure 4, Figure 5, Figure 6). Results demonstrated superior anti-tumor effect when USMB and XRT were combined compared to the single modality treatments [80].

In an additional study using MRI-guided FUS (MRgFUS), New Zealand White rabbits were used to host PC-3 xenografts on the hind legs [83]. USMB treatment consisted of a bolus injection of microbubbles (Definity), followed by sonication for 14 min using an MRgFUS system (Sonalleve, Profound Medical). Sonication was focused on a 10–20 mm in diameter circular planning target volume. Tumor localization was dependent on the size of the tumors, and of T1 and T2-weighed MRI scans on a 3T system (Achieva, Philips Healthcare). Radiation treatments consisted of whole tumor exposure to an 8 Gy dose of radiation either alone or in combination with USMB treatment. Radiation was delivered immediately after USMB exposure. After 24 h post-therapy, immunohistochemistry analysis observing tumor morphology (hematoxylin and eosin; H&E) and cell death (terminal deoxynucleotidyl transferase dUTP nick end labeling (TUNEL)) was conducted. Positive TUNEL staining corroborated the cell destruction response areas on respective H&E stains. Combined treatments (17.9% ± 5.7%) showed greater areas of cell death which were more diffuse across the tumor compared to XRT- only (7.6% ± 3.8%) or USMB-only (5.7% ± 2.5%) treatments indicating a radiation enhancement effect using USMB therapy in large tumor models [83].

## USMB and XRT effects on cell membrane

**Figure 7:**
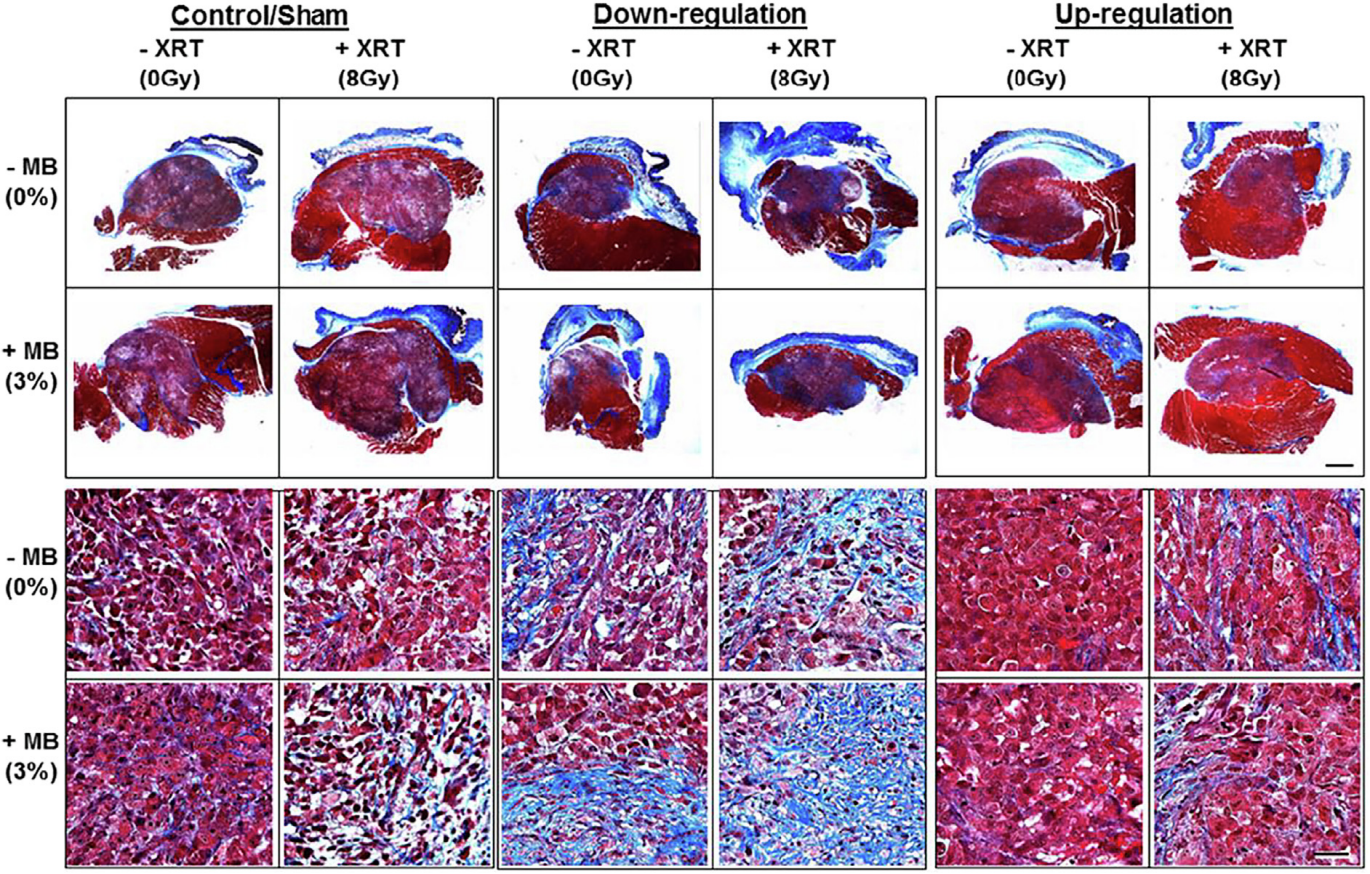
Tumor sections labeled for Masson trichrome staining. Different colors indicate collagen fibers (blue), cytoplasm and muscles (red), and nuclei (black). Groups included untreated tumors (control), ultrasound-stimulated microbubbles (USMB; 3%), radiation therapy (XRT, 8Gy), and combined treatments (USMB + 8Gy). The first and second row displays representative low-magnification images of trichrome staining. The scale bar represents 1 mm (B) Third and fourth row displays representative high-magnification images of trichrome staining. The scale bar represents 50 μm. Results from high magnification microscopy images revealed higher fibrotic tissues in down-regulated tumors compared to up-regulated tumor tissue. From [86].

It has now been well established that damage to the endothelial cell membrane causes secondary tumor cell death [85]. A recent study supports the involvement of cell membrane-metabolism-related pathways, including an up-regulation of UDP glycosyltransferase 8 (UGT8) in the apoptotic signaling cascade [86]. UGT8 is known to catalyze the transfer of galactose to ceramide, a lipid molecule that promotes apoptosis. The study examined the role of UGT8 in the response of prostate tumors to USMB radiation enhancement therapy. Experiments were conducted with UGT8 levels up-regulated or down-regulated in both *in vitro* and *in vivo*. In the latter, xenograft tumors generated from stably transfected PC3 cells were treated with USMB, XRT, or USMB + XRT. Greater cellular damage was seen in tumors with down-regulated UGT8 in comparison with control tumors. In contrast, tumors with upregulated UGT8 had lesser damage than control tumors. Power Doppler and photoacoustic imaging showed a reduction in the vascular index and oxygen saturation, respectively, with UGT8 down-regulation. The down-regulation of UGT8 corresponded with an increase in ceramide accumulation leading to more cell death, which resulted in a greater enhancement of radiation effect as a result of USMB-mediated vascular disruption [86] (Fig. 7).

## Involvement of ceramide pathway *in vitro*

Understanding the molecular mechanisms in which USMB interacts with endothelial cells is crucial to better characterize and optimize treatments. Investigations involving HUVEC were conducted to observe the effects of USMB only, XRT only, or combination treatment (USMB + XRT) [26]. The effects of treatment on cells were evaluated at 0, 3, 6, and 24 h after treatment. Treatments were delivered alongside sphingolipid-based signaling modulators, including ceramide, fumonisin-B1, monensin, and S1P. Treatment responses were assessed using immunohistopathology, clonogenic survival methods, immunofluorescence, electron microscopy, and endothelial cell tube-forming assays (the assay measures the ability of endothelial cells to form capillary-like structures (tubes). The results showed that there was a lower number of surviving cells in the USMB + XRT combined treatment group than in either of the USMB or XRT treatments alone. In S1P-only treated endothelial cells, USMB + XRT treatments reduced the capacity to form tubes. However, the combined treatments did not affect tube- formation when treated with either the fumonisin B1- or monensin. In summary, these results suggest the role of ceramide signaling as a key player in cell death initiation following USMB + XRT treatments [26].

## *In vitro* radiosensitization effect of USMB

Several studies, primarily *in vitro*, have recently been conducted in investigating the effect of USMB and radiation on various tumor cell types including metastatic follicular thyroid carcinoma cells (FTC-238), non-small cell lung carcinoma cells (NCI-H727) with kV and MV energies [75], esophageal squamous cell carcinoma cell lines (KYSE-410, KYSE-1140, KYSE-510) [60], [61], [87], and ovarian cancer [88]. In these studies, a significant increase in tumor cell death was observed with the combination of USMB treatments alongside XRT compared to either modality alone. Additionally, work conducted by Deng *et al*. with nasopharyngeal carcinoma cells (CNE-2) corroborated a similar effect in an *in vivo* and *in vitro* studies. The vascular integrity in nude mice xenograft models hosting CNE-2 tumors was observed using color Doppler flow imaging, followed by immunohistochemistry analysis of cell survival. Tumors exposed to USMB mildly reduced blood flow and CD34 expression, with increased tumor cell death, and also had notable effects on angiogenic marker expression. All of this evidence corroborates the enhancing effect that USMB has when applied alongside radiation [89].

## Involvement of ceramide pathway *in vivo*

Past works have extensively investigated the role of the ceramide signaling pathway and other signaling molecules in response to USMB treatments using tumor models *in vivo* [81], [90]. The use of vascular-altering biological agents was shown to be capable of enhancing ceramide-driven anti-vascular response [91]. In another study, tumor response to USMB and XRT was assessed 24 h after treatment [85]. Immunohistochemical analysis using ISEL and TUNEL was used to observe levels of cell death. The results demonstrated increased cell death in tumors after treatment with USMB + XRT relative to untreated tumors or tumors treated by XRT alone. Furthermore, several biomarkers were investigated to evaluate responses of tumor cells and extracellular components including cell proliferation (Ki67), vascular leakage (factor VIII), angiogenesis (CD31), ceramide formation, angiogenesis (vascular endothelial growth factor (VEGF)), hypoxia (prolyl hydroxylase domain protein 2 (PHD2)), and DNA damage (γH2AX). Results demonstrated that treatments with USMB and XRT resulted in reduced vascularity and an elevation in ceramide production alongside, increased DNA damage and fewer proliferating tumor cells. Furthermore, a reduction in tumor oxygenation was also detected, corroborating the effects of vascular disruption in altering the tumor microenvironment [85].

## Mechano-acoustic activation of the ASMase-ceramide pathway by USMB

**Figure 8:**
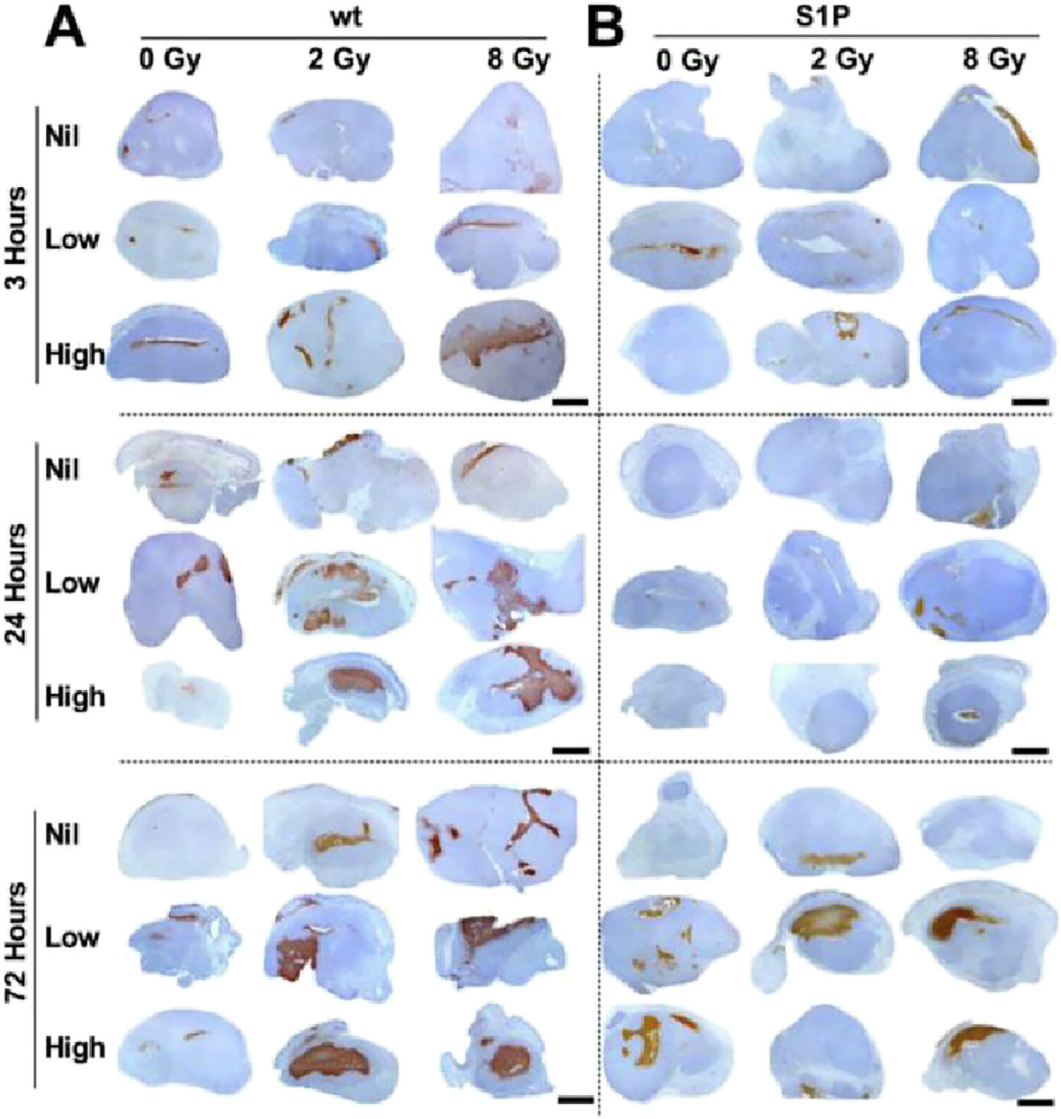

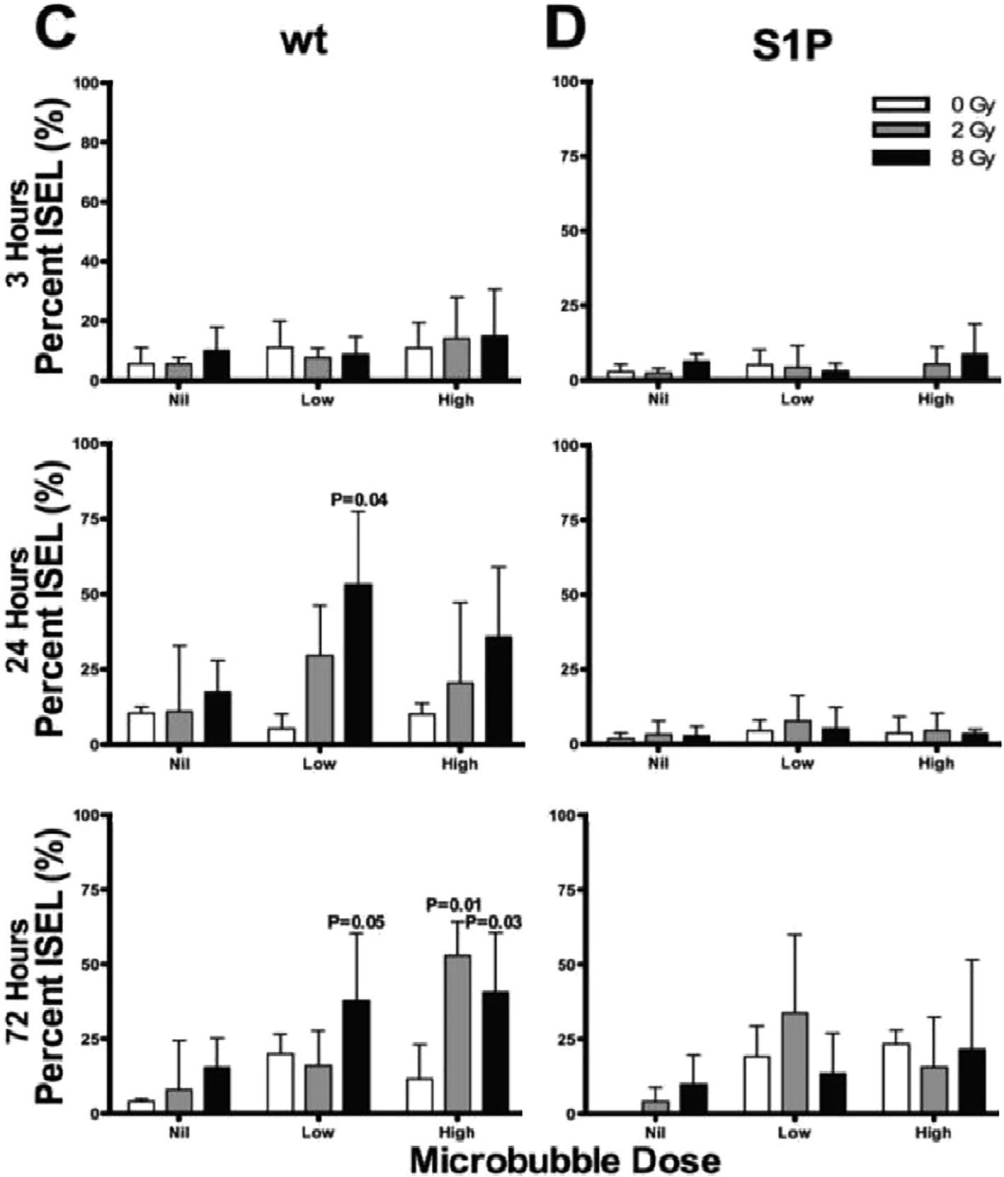
Acid sphingomyelinase (ASMase) and mecho-acoustical effects: Tumor cell death response assessments. Representative low magnification views of in situ end labeling (ISEL) staining obtained from (A) tumor cross-sections from wild-type (wt) and (B) tumor cross-sections from sphingosine-1-phosphate (S1P)-treated animals. Mice were treated with ultrasound-stimulated microbubbles (USMB; low is 1%, and high is 3% volume of total blood volume [v/v]) and radiation. The scale bar indicates 1 mm. Quantitative analysis of cell death in (C) wt and (D) S1P-treated mice. Statistical analysis tested using one way ANOVA followed by two-sided Tukey’s Honest test indicated significant increase in cell death in wt mice at 24 and 72 h, while no such increase was observed in animals treated with S1P. P value presented as* for < 0.05 are depicted in graphs. Comparison was made comparing different treatment groups to the control (0 Gy, 0% USMB (Nil)). The error bars represent the standard deviation (SD). From [22].

Ceramide is known to be a key component in facilitating vascular related changes in response to radiation [22], [86], [92], [93]. Recent experimentation has looked to find other important components which work in synergy with ceramide. The ASMase-ceramide pathway in particular has previously demonstrated a role in USMB-based enhancement of radiation [22]. The mechano-acoustic activation of the ASMase-ceramide pathway by USMB was evaluated using genetic and chemical methods. Wild-type and ASMase knockout (KO) mice were implanted with fibrosarcoma xenografts (MCA-129). A cohort of wild-type mice received S1P treatments prior to USMB and XRT. Mice were treated with varying concentrations of microbubbles (0%, 1%, 3%) followed by exposure to different radiation doses (2 or 8 Gy). Quantitative 3D Doppler ultrasound and immunohistochemistry was used to evaluate treatment response at baseline, and at 3, 24, and 72 h after treatment. Results demonstrated a decrease in tumor perfusion of up to 46% by 3 h following radiation and USMB, confirming the significant effect with USMB and XRT at 24 h (P < .001). The peak of this effect was observed at 24 h that persisted for up to 72 h. This was further accompanied by extensive tumor cell death. In contrast, S1P-treated and ASMase KO mice for all treatment conditions showed minimal tumor responses and changes were noted to be statistically non-significant [22]. The study further confirmed that the enhanced radiation response results from acute vascular shutdown that takes place following radiation doses (<8 Gy) prior to USMB exposure [22] (Fig. 8).

**Figure 9:**
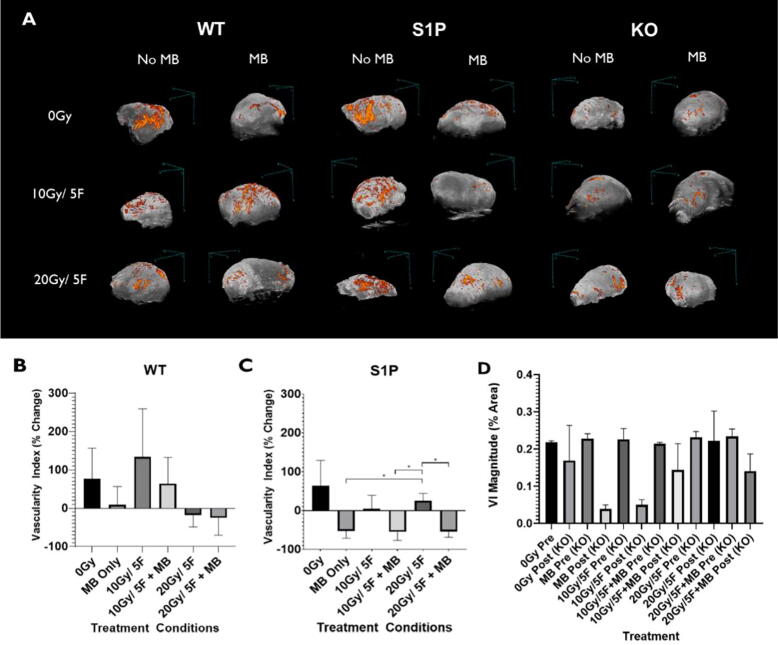
Power Doppler analysis of vascular changes in tumors as a result of Acid sphingomyelinase (ASMase) effects. (A) Maximum intensity projections of power Doppler signals within a 3D volumetric scan of whole tumors undergoing respective treatments. The color bar represents a range from 11dB (black) to 30dB (orange). Tumors underwent fractionated XRT alone or combined with ultrasound-stimulated microbubbles (USMB) treatments. Scans were collected 72 h after the final treatment day. (B), (C), and (D) Quantified vascular index percent change of signal in each treatment condition of wild-type (WT), S1P, and ASMase knockout groups. Error bars represent the standard error of the mean (SEM). All data underwent statistical analysis by Welch’s t-test, *p < 0.05. Preliminary data from [81].

Most studies evaluating the effects of USMB + XRT have only used single doses of radiation and reveal crucial information on the effects of single fraction interactions [21], [27], [94]. However, few studies have looked at long-term exposures of fractionated XRT alongside USMB and tried understanding how the effects scale in a more clinically relevant regimen [80], [81]. For this, ASMase KO and wild-type mice were implanted with MCA-129 tumors on the hind leg and treated with radiation regimens of 10 Gy/5 Fractions (2 Gy daily for 5 days) or 20 Gy/5 fractions (4 Gy daily for 5 days) [81]. These radiation treatment regimens were delivered combined with or without USMB treatment delivered twice weekly. In addition, another cohort of wild-type mice was pre-treated with S1P. Immunohistochemical analysis was used to observe changes in cell death (Caspase-3 and TUNEL), microvascular density changes (CD31), and cell proliferation (Ki-67) within tumors, 72 h post-treatment. Results showed addition of USMB alongside 10 Gy/5 fractions was capable of enhancing cell death and vascular damage effects to a level similarly seen in the wild-type animals 4 Gy daily XRT (20 Gy/5 fractions) alone cohort. S1P treated mice exhibited a radioresistant phenotype when exposed to fractionated XRT or USMB treatments alone. However, when combined with USMB, fractionated XRT treatments were capable of overcoming the radioresistant effects of S1P. This was not reflected in the ASMase KO cohorts, in which the addition of USMB was not capable of overcoming the radioresistant effect [74], [81] (Fig. 9).

## USMB and radiation with nanobubbles

Microbubbles have been thoroughly investigated in their applications. However, these bubble sizes are defined strictly in the magnitude of microns. Bubbles of smaller diameters less than 200 nanometers (nm), nanobubbles, have recently been of interest in being applied therapeutically [95], [96], [97], [98]. Studies investigating the radioenhancing effects of ultrasonically-stimulated nanobubbles have been carried out [82], [99]. Because of their size, these particles are not confined to the vasculature like microbubbles are, and permeate into tumor interstitia [100], [101]. *In vivo* experiments using combined ultrasound-stimulated nanobubbles (USNB) and radiation treatments were conducted on mice bearing human prostate cancer (PC3) tumors and were compared against conventional USMB and radiation alone (single 8 Gy fraction, XRT) [82]. Photoacoustic imaging was used to monitor oxygenation levels and the effects of treatments non-invasively, followed by histological examination. Compared to controls, a 20% decrease in oxygenation was observed using photoacoustic metrics of oxygen saturation in USNB treated tumors 24 h after treatment. A significant enhancement of treatment effect was observed when using nanobubbles compared to the effect in microbubbles treated groups. Treatment with USMB alone and XRT alone resulted in 7% ± 2% and 9% ± 6% cell death, respectively, whereas treatment with USNB alone resulted in 20% ± 6% cell death. Furthermore, the combination of USNB + XRT resulted a greater cell death effect of 40% ± 5% compared to 15% ± 3% cell death for USMB + XRT [82]. Separate from this work, other preparatory studies have been conducted to characterize nanobubbles and their behavior [102], [103], [104], [105], [106], [107].

## USMB and XRT in human treatments

**Figure 10:**
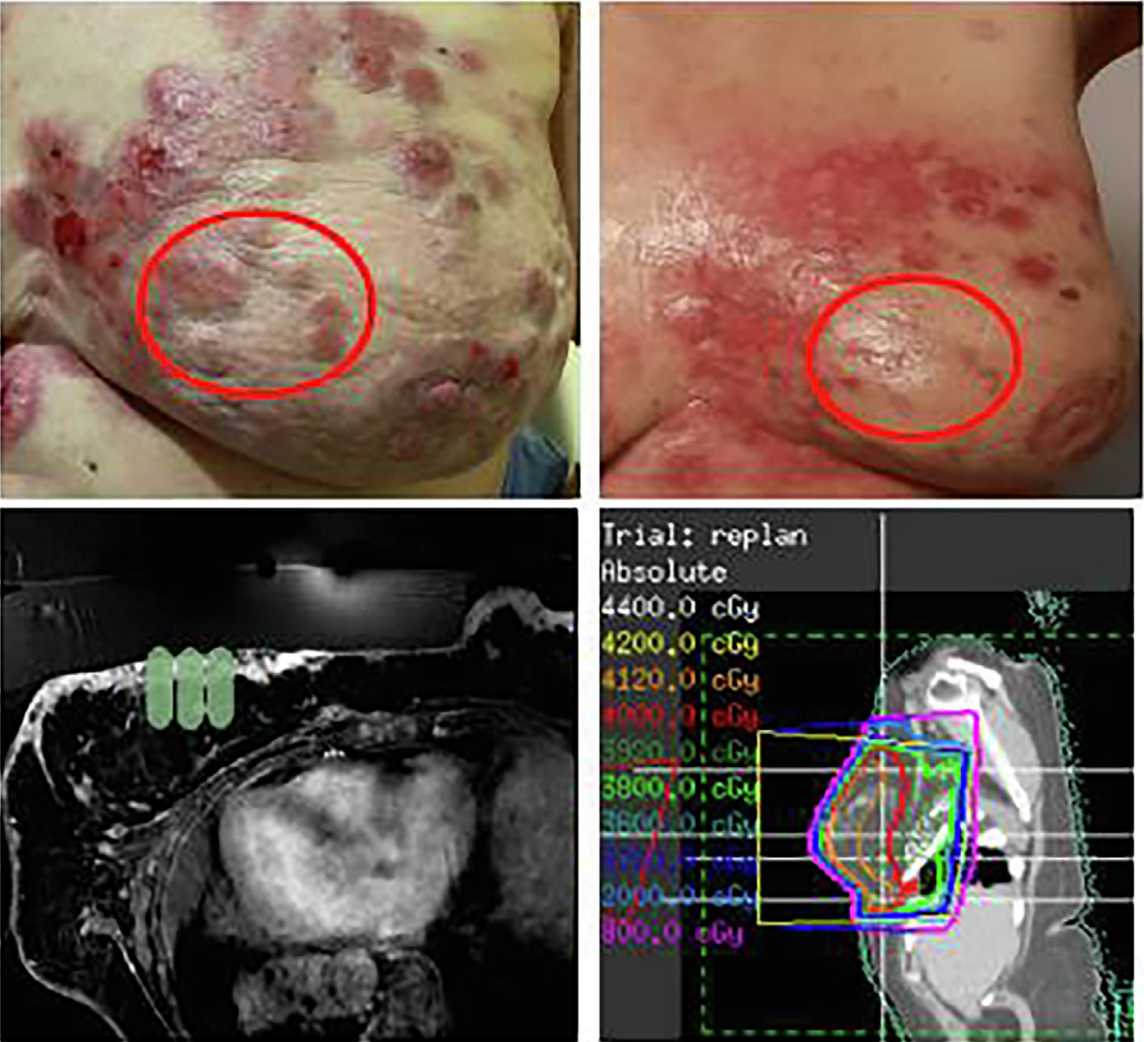
Pilot data from phase I study of ultrasound-microbubble stimulation in breast cancer. Top left: Clinical view of treated lesions before treatment (red ellipse) with MRg-FUS + MB + XRT for radiation enhancement. Ellipse is 5.0 cm in the largest dimension. Top right: Clinical view of lesions indicating diminished lesion sizes 4 weeks after treatment with MRg-FUS + MB. Bottom left: MRI plan indicating where focused ultrasound was directed for microbubble stimulation. Bottom right: MRI plan indicating where radiation was directed for treatment. Treatment with radiation was administered immediately after MRg-FUS + MB therapy From [84].

**Figure 11:**
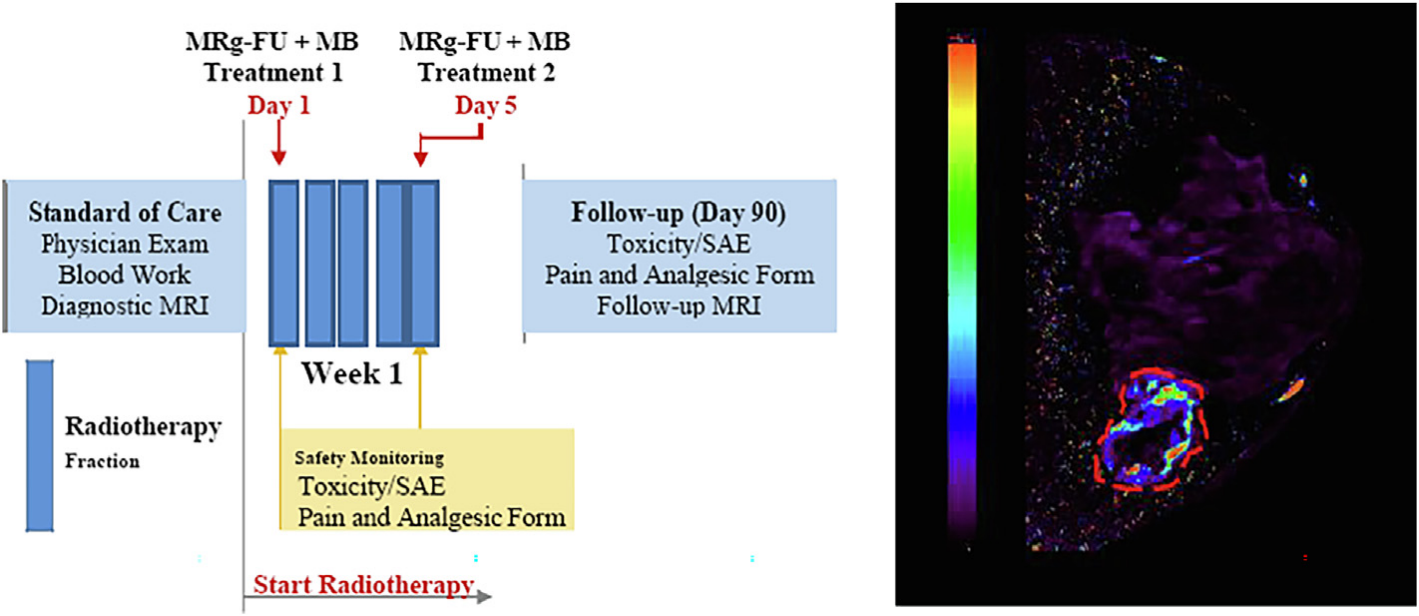
(Left) Treatment outlines. Fractionation regimens: Two (2) MRg-FUS + MB treatments are scheduled as indicated. There will be two permitted stratifications for radiation dose: 2000 cGy/5 fractions. (Right) Preliminary DCE MRI K-Trans Data. The dashed line indicates a tumor. The color bar indicates relative DEC k-trans parameter data From [84].

Clinical trials have recently been conducted to implement the use of USMB alongside existing clinical treatment regimen and image guided therapies [108], [109], [84]. A pilot first-in-human study is underway using a first generation Sonalleve device (Philips Healthcare/Profound Medical Systems) with a target of 20 patients (10 breast cancer and 10 head and neck patients) to be recruited using MRgFUS [84]. Definity microbubbles were used in this study. Preliminary results in patients with breast cancer have demonstrated a promising response in lesions targeted by MRgFUS + XRT treatments (see Figure 10, Figure 11) (Table 1) with follow-up results for one year available. Patients received doses of radiation ranging from 2000 cGy/5 fractions to 4000 cGy/10 fractions. All patients received 2 treatments of MRg-FUS + MB on days 1 and 5 of their XRT radiotherapy regimens. Results from the breast cancer pilot study indicate that 9/10 tumors show complete response after USMB + XRT using an MRg FUS system. This was seen after 12 months of follow-up for each patient, in which either complete resolution of tumor or replacement fibrosis with no tumor regrowth. In patients where the replacement fibrosis was observed in place of the treated tumor, no evidence of disease recurrence was observed following one year after treatment. See Figure 10, Figure 11 [84] .

**Table 1:** Treatment details and tumor volumes at various times after treatment. Green indicates a complete response. Red indicates disease progression From [84].

Other radiation-enhancing clinical research has taken place using transarterial radioembolization (TARE) combined with USMB in participants with hepatocellular carcinoma (HCC) [33]. Participants who were scheduled for TARE treatments were randomized into one of two groups; TARE alone or TARE with ultrasound-triggered microbubbles destruction (delivered 1-4 h and approximately 1 and 2 weeks after TARE). Differences across Response Evaluation Criteria in Solid Tumors (mRECIST) reads and prevalence of tumor response were compared by using a Mann-Whitney U test, and Fisher exact test, respectively. The differences in time required for the next treatment and overall survival curves were compared by using a log-rank (Mantel-Cox) test. A greater prevalence of tumor response (14 of 15 [93%; 95% CI: 68, 100] vs five of 10 [50%; 95% CI: 19, 81]; P = .02) was shown in preliminary efficacy results in participants who underwent both ultrasound-triggered microbubbles destruction and TARE (P = .02) and appeared to result in improved hepatocellular carcinoma treatment response [33].

## Ultrasound and microbubbles mediated immune response

Tumor cells are usually found to express some harmful immunosuppressive substances that weaken the body's immune system [110]. In cancer patients, the immune system is stimulated using varieties of substances (made by the body) that recognize cancer cells and attack them. This treatment is known as immunotherapy [111], [112]. At present different types of cancers are treated using several types of immunotherapy substances including checkpoint inhibitors, immune system modulators, monoclonal antibodies, T-cell transfer therapy, and treatment vaccines [113], [114], [115], [116], [117], [118], [119], [120], [121], [122], [123], [124], [125], [126]. The therapeutic effectiveness and cost of the immunotherapy remain questionable as patients treated with the abovementioned substances may have severe side effects with substances being poorly hydrophilic and easily degraded in the blood circulation [127], [128], [129]. In past few years, it has been found that ultrasound can be used to stimulate an anti-tumor immune response [130], [131], [132], [133]. Ultrasound on its own can regulate the immune response by thermally ablating the tumors however, when used alongside immunotherapeutics it helps in increasing the therapeutic efficacy of immunotherapy [134], [135], [136]. Using ultrasound can help in the precise ablation of tumors and reduce the required drug dosage needed to treat cancer thus resulting in minimal side effects [137]. Different forms of ultrasound techniques including high-intensity focused ultrasound (HIFU), low-intensity focused ultrasound (LIFU), ultrasound-targeted microbubble destruction (UTMD), and sonodynamic therapy (SDT) can be used in combination with immunotherapy to boost the immune system of cancer patients [136], [138], [139], [140], [141], [142], [143], [144], [145], [146], [147], [148], [149], [150], [151]. Recently, a technique of microbubble-assisted ultrasound-guided immunotherapy has gained immense attention by demonstrating improved anti-tumor immunity [152], [153]. This method utilizes the delivery of an immunotransmitter-cyclic guanosine monophosphate-adenosine monophosphate (cGAMP). This is done by combining nucleotide nanocomplexes into the microbubbles (integrating microbubbles with nanocomplexes) [152], [154]. The complex of nanocomplexes and microbubbles is delivered into antigen-presenting cells (APCs), where the microbubbles release cGAMP that leads to activation of GMP-AMP synthase (cGAS)-stimulator of interferon genes (STING) pathway stimulating type I interferon responses that are pivotal for tumor-specific T cells priming [152], [155]. A preclinical study conducted by Li *et al.* demonstrated that using the technique of ultrasound-guided immunotherapy of cancer they were able to activate the systemic anti-tumor immunity in mouse-bearing mammary breast carcinoma 4T1 that resulted in metastasis inhibition in breast cancer. Their results revealed a combination of ultrasound-guided immunotherapy and an anti-PD-1 antibody resulted in a 76% median survival increase in animals along with pulmonary metastatic nodules reduced to approximately 60% as compared to the animals that were given either of the treatment alone [152]. This findings provided a promising future for treating cancer patients with tumor metastasis.

In addition to this, ultrasound-mediated abscopal effects have also been reported in some studies [156], [157]. Experiments conducted with murine models of melanoma and hepatocellular carcinoma demonstrated infiltration of the immune cell following histotripsy [157]. Inhibition in the growth of pulmonary metastases was observed at a site of tumors that remained untreated. The stimulation of immune response following histotripsy was found to be linked with the translocation of calreticulin to the cell membrane with local release of intratumoral high mobility group box protein 1 [157]. Another study by Hu *et al.* reported that ultrasound and nanobubbles mediated a systemic immune response and abscopal effect when combined with immunotherapy (anti-PD-1 antibody) [158]. It was seen that the mouse models of RM1 (prostate cancer), MC38 (colon cancer), and B16 (melanoma) xenograft resulted in an increase in antigen release and enhancement in the number of innate immune cells (APCs, in the tumor microenvironment and draining lymph nodes). Additionally, a group of mice implanted with tumors on both legs showed similar growth patterns on both sides even though only one leg tumor was exposed to USNB + anti-PD1, while the other side remained unexposed. This result demonstrated the ability of USNB to trigger systemic antitumor effects and abscopal effects [158]. Together, these findings suggest that ultrasound, when used alongside immunotherapy, can enhance the anti-tumor effects. Despite the success of immunotherapy in preclinical studies, its translation in clinical settings remains challenging. Few clinical trials have reported short-term and long-term survival benefits in patients with advanced melanoma [159], [160]. However, trials conducted with patient of renal cell carcinoma [161], non-small-cell lung cancer [162], small-cell lung cancer [163] and prostate cancer [164] showed less prominent results. In clinical settings, the treatment of cancer with immunotherapies might be tumor specific.

Furthermore radiation combined with immunotherapy has also shown promising outcomes in both preclinical and clinical studies demonstrating systemic immune response and abscopal effect [165], [166], [167], [168], [169]. However, no study to our knowledge has yet been carried out looking at the immune response and abscopal effect combining ultrasound, microbubbles, and radiation. Since radiation on its own is known to induce these effects [170], [171], [172], [173], [174], [175], [176], it might be interesting to see if an enhanced immunostimulatory response occurs by combining radiation with ultrasound and microbubbles.

## Clinical implications and summary

USMB therapy can enhance radiation effects by damaging tumor blood vessels. The combinatory impact of USMB and radiation-mediated endothelial cellular perturbations leading to secondary tumor cell death has been documented in several *in vitro* and *in vivo* studies. USMB elucidates benefits over conventional cancer surgical treatments by spatially confining ultrasound energy within the tumor volume. Combining USMB with a lower dose of radiation is demonstrated to elicit similar vascular endothelial damage as seen using a single high dose of XRT. This combined technique can spare healthy tissues and minimize systemic toxicity. Few clinical studies have also seen fruitful results regarding the safety and feasibility of USMB. Thus, this technique has the advantages of high precision that holds a promising future in treating cancer patients. Lastly, more studies should be conducted looking at the combinatory immune effects of USMB and radiation treatment to better understand the role of the immune system in cancer development.

## Declaration of Competing Interest

The authors declare that they have no known competing financial interests or personal relationships that could have appeared to influence the work reported in this paper.

## References

[1] Hall E.J., Giaccia A.J. (2012).

[2] Baskar R., Lee K.A., Yeo R., Yeoh K.W. (2012). Cancer and radiation therapy: Current advances and future directions. Int J Med Sci.

[3] Baskar R., Dai J., Wenlong N., Yeo R., Yeoh K.-W. (2014). Biological response of cancer cells to radiation treatment. Front Mol Biosci.

[4] Paris F., Fuks Z., Kang A., Capodieci P., Juan G., Ehleiter D. (2001). Endothelial apoptosis as the primary lesion initiating intestinal radiation damage in mice. Science (80-).

[5] Peña L.A., Fuks Z., Kolesnick R.N. (2000). Radiation-induced apoptosis of endothelial cells in the murine central nervous system: protection by fibroblast growth factor and sphingomyelinase deficiency. Cancer Res.

[6] Garcia-Barros M., Paris F., Cordon-Cardo C., Lyden D., Rafii S., Haimovitz-Friedman A. (2003). Tumor Response to Radiotherapy Regulated by Endothelial Cell Apoptosis. Science (80-).

[7] Folkman J., Camphausen K. (2001). Cancer: What does radiotherapy do to endothelial cells?. Science.

[8] Kolesnick R., Fuks Z. (2003). Radiation and ceramide-induced apoptosis. Oncogene.

[9] Moeller B.J., Cao Y., Li C.Y., Dewhirst M.W. (2004). Radiation activates HIF-1 to regulate vascular radiosensitivity in tumors: Role of reoxygenation, free radicals, and stress granules. Cancer Cell.

[10] Moeller B.J., Dreher M.R., Rabbani Z.N., Schroeder T., Cao Y., Li C.Y. (2005). Pleiotropic effects of HIF-1 blockade on tumor radiosensitivity. Cancer Cell.

[11] Santana P., Peña L.A., Haimovitz-Friedman A., Martin S., Green D., McLoughlin M. (1996). Acid Sphingomyelinase-Deficient Human Lymphoblasts and Mice Are Defective in Radiation-Induced Apoptosis. Cell.

[12] Sathishkumar S., Boyanovsky B., Karakashian A.A., Rozenova K., Giltiay N.V., Kudrimoti M. (2005). Elevated sphingomyelinase activity and ceramide concentration in serum of patients undergoing high dose spatially fractionated radiation treatment. Implications for endothelial apoptosis. Cancer Biol Ther.

[13] Siemann D.W., Horsman M.R. (2004). Targeting the tumor vasculature: A strategy to improve radiation therapy. Expert Rev Anticancer Ther.

[14] Cairns R., Papandreou I., Denko N. (2006). Overcoming physiologic barriers to cancer treatment by molecularly targeting the tumor microenvironment. Mol Cancer Res.

[15] Fukumura D., Jain R.K. (2007). Tumor microvasculature and microenvironment: Targets for anti-angiogenesis and normalization. Microvasc Res.

[16] Graham K., Unger E. (2018). Overcoming tumor hypoxia as a barrier to radiotherapy, chemotherapy and immunotherapy in cancer treatment [Internet]. Int J Nanomed.

[17] Olivera A., Spiegel S. (2001). Sphingosine kinase: A mediator of vital cellular functions. Prostaglandins Other Lipid Mediat.

[18] Hla T. (2003). Signaling and biological actions of sphingosine 1-phosphate. Pharmacol Res.

[19] Leclercq T.M., Pitson S.M. (2006). Cellular signalling by sphingosine kinase and sphingosine 1-phosphate.

[20] Wang P., Yuan Y., Lin W., Zhong H., Xu K., Qi X. (2019). Roles of sphingosine-1-phosphate signaling in cancer. Cancer Cell Int.

[21] Czarnota G.J., Karshafian R., Burns P.N., Wong S., Al Mahrouki A., Lee J.W. (2012). Tumor radiation response enhancement by acoustical stimulation of the vasculature. Proc Natl Acad Sci U S A.

[22] El Kaffas A., Al-Mahrouki A., Hashim A., Law N., Giles A., Czarnota G.J. (2018). Role of acid sphingomyelinase and ceramide in mechano-acoustic enhancement of tumor radiation responses. J Natl Cancer Inst.

[23] Kolesnick R. (2002). The therapeutic potential of modulating the ceramide/sphingomyelin pathway. J Clin Invest.

[24] Nofiele J.I.T., Karshafian R., Furukawa M., Al Mahrouki A., Giles A., Wong S. (2013). Ultrasound-activated microbubble cancer therapy: Ceramide production leading to enhanced radiation effect in vitro. Technol Cancer Res Treat.

[25] Al-Mahrouki A.A., Karshafian R., Giles A., Czarnota G.J. (2012). Bioeffects of Ultrasound-Stimulated Microbubbles on Endothelial Cells: Gene Expression Changes Associated with Radiation Enhancement In Vitro. Ultrasound Med Biol.

[26] Al-Mahrouki A.A., Wong E., Czarnota G.J. (2015). Ultrasound-stimulated microbubble enhancement of radiation treatments: Endothelial cell function and mechanism. Oncoscience.

[27] Tran W.T., Iradji S., Sofroni E., Giles A., Eddy D., Czarnota G.J. (2012). Microbubble and ultrasound radioenhancement of bladder cancer. Br J Cancer.

[28] Caissie A., Karshafian R., Hynynen K., Czarnota G.J. (2011). Nanoimaging.

[29] Tarapacki C., Lai P., Tran W.T., El Kaffas A., Lee J., Hupple C. (2016). Breast tumor response to ultrasound mediated excitation of microbubbles and radiation therapy in vivo. Oncoscience.

[30] Kwok S.J.J., El Kaffas A., Lai P., Al Mahrouki A., Lee J., Iradji S. (2013). Ultrasound-Mediated Microbubble Enhancement of Radiation Therapy Studied Using Three-Dimensional High-Frequency Power Doppler Ultrasound. Ultrasound Med Biol.

[31] Oeffinger B.E., Vaidya P., Ayaz I., Shraim R., Eisenbrey J.R., Wheatley M.A. (2019). Preserving the Integrity of Surfactant-Stabilized Microbubble Membranes for Localized Oxygen Delivery. Langmuir.

[32] Daecher A., Stanczak M., Liu J.-B., Zhang J., Du S., Forsberg F. (2017). Localized microbubble cavitation-based antivascular therapy for improving HCC treatment response to radiotherapy. Cancer Lett.

[33] Eisenbrey J.R., Forsberg F., Wessner C.E., Delaney L.J., Bradigan K., Gummadi S. (2021). US-triggered Microbubble Destruction for Augmenting Hepatocellular Carcinoma Response to Transarterial Radioembolization: A Randomized Pilot Clinical Trial. Radiology.

[34] El Kaffas A., Czarnota G.J. (2015). Biomechanical effects of microbubbles: From radiosensitization to cell death. Future Oncol.

[35] Czarnota G.J. (2015). Ultrasound-stimulated microbubble enhancement of radiation response. Biol Chem.

[36] Lacerda Q., Tantawi M., Leeper D.B., Wheatley M.A., Eisenbrey J.R. (2021). Emerging Applications of Ultrasound-Contrast Agents in Radiation Therapy. Ultrasound Med Biol.

[37] Schutt E.G., Klein D.H., Mattrey R.M., Riess J.G. (2003). Injectable microbubbles as contrast agents for diagnostic ultrasound imaging: The key role of perfluorochemicals. Angew Chem – Int Ed.

[38] Dijkmans P.A., Juffermans L.J.M., Musters R.J.P., van Wamel A., ten Cate F.J., van Gilst W. (2004). Microbubbles and ultrasound: From diagnosis to therapy. Eur J Echocardiogr.

[39] Hernot S., Klibanov A.L. (2008). Microbubbles in ultrasound-triggered drug and gene delivery. Adv Drug Deliv Rev.

[40] Simpson D.H., Burns P.N., Averkiou M.A. (2001). Techniques for perfusion imaging with microbubble contrast agents. IEEE Trans Ultrason Ferroelectr Freq Control.

[41] Jugniot N., Bam R., Meuillet E.J., Unger E.C., Paulmurugan R. (2021). Current status of targeted microbubbles in diagnostic molecular imaging of pancreatic cancer. Bioeng Transl Med.

[42] Battaglia V., Cervelli R. (2017). Liver investigations: Updating on US technique and contrast-enhanced ultrasound (CEUS). Eur J Radiol.

[43] Mehta K.S., Lee J.J., Taha A.A., Avgerinos E., Chaer R.A. (2017). Vascular applications of contrast-enhanced ultrasound imaging. J Vasc Surg.

[44] Huang D.Y., Yusuf G.T., Daneshi M., Ramnarine R., Deganello A., Sellars M.E. (2018). Contrast-enhanced ultrasound (CEUS) in abdominal intervention. Abdominal. Radiology.

[45] Dietrich C.F., Nolsøe C.P., Barr R.G., Berzigotti A., Burns P.N., Cantisani V., Guidelines and Good Clinical Practice Recommendations for Contrast-Enhanced Ultrasound (CEUS) in the Liver-Update (2020). WFUMB in Cooperation with EFSUMB, AFSUMB, AIUM, and FLAUS: WFUMB in Cooperation with EFSUMB, AFSUMB, AIUM and FLAUS. Ultrasound Med Biol.

[46] Rix A., Curaj A., Liehn E., Kiessling F. (2020). Ultrasound Microbubbles for Diagnosis and Treatment of Cardiovascular Diseases. Semin Thromb Hemost.

[47] Sridharan A., Eisenbrey J.R., Forsberg F., Lorenz N., Steffgen L., Ntoulia A. (2021). Ultrasound contrast agents: microbubbles made simple for the pediatric radiologist. Pediatr Radiol.

[48] Qin S., Caskey C.F., Ferrara K.W. (2009). Ultrasound contrast microbubbles in imaging and therapy: physical principles and engineering. Phys Med Biol.

[49] Cheng M., Li F., Han T., Yu A.C.H., Qin P. (2019). Effects of ultrasound pulse parameters on cavitation properties of flowing microbubbles under physiologically relevant conditions. Ultrason Sonochem.

[50] Lin Y., Lin L., Cheng M., Jin L., Du L., Han T. (2017). Effect of acoustic parameters on the cavitation behavior of SonoVue microbubbles induced by pulsed ultrasound. Ultrason Sonochem.

[51] Chowdhury S.M., Abou-Elkacem L., Lee T., Dahl J., Lutz A.M. (2020). Ultrasound and microbubble mediated therapeutic delivery: Underlying mechanisms and future outlook. J Control Release.

[52] Collis J., Manasseh R., Liovic P., Tho P., Ooi A., Petkovic-Duran K. (2010). Cavitation microstreaming and stress fields created by microbubbles. Ultrasonics.

[53] Guo G., Ma Y., Guo Y., Zhang C., Guo X., Tu J. (2017). Enhanced porosity and permeability of three-dimensional alginate scaffolds via acoustic microstreaming induced by low-intensity pulsed ultrasound. Ultrason Sonochem.

[54] Meng L., Liu X., Wang Y., Zhang W., Zhou W., Cai F. (2019). Sonoporation of Cells by a Parallel Stable Cavitation Microbubble Array. Adv Sci.

[55] Tan C., Yan B., Han T., Yu A.C.H., Qin P. (2022). Improving temporal stability of stable cavitation activity of circulating microbubbles using a closed-loop controller based on pulse-length regulation. Ultrason Sonochem.

[56] Bailey M.R., Khokhlova V.A., Sapozhnikov O.A., Kargl S.G., Crum L.A. (2003). Physical mechanisms of the therapeutic effect of ultrasound (a review). Acoust Phys.

[57] Kimmel E. (2006). Cavitation bioeffects. Crit Rev Biomed Eng.

[58] Ohl S.W., Klaseboer E., Khoo B.C. (2015). Bubbles with shock waves and ultrasound: A review. Interface Focus.

[59] Luo J., Niu Z. (2019). Jet and Shock Wave from Collapse of Two Cavitation Bubbles. Sci Rep.

[60] Fan Z., Chen D., Deng C.X. (2014). Characterization of the dynamic activities of a population of microbubbles driven by pulsed ultrasound exposure in sonoporation. Ultrasound Med Biol.

[61] He Y., Yu M., Wang J., Xi F., Zhong J., Yang Y. (2020). Improving the Therapeutic Effect of Ultrasound Combined With Microbubbles on Muscular Tumor Xenografts With Appropriate Acoustic Pressure. Front Pharmacol.

[62] Liu H.-L., Hsieh H.-Y., Lu L.-A., Kang C.-W., Wu M.-F., Lin C.-Y. (2012). Low-pressure pulsed focused ultrasound with microbubbles promotes an anticancer immunological response. J Transl Med.

[63] Sorace A.G., Hoyt K. (2014). Imaging the microvascular response to ultrasound-stimulated therapy in a preclinical animal model of breast cancer. IEEE Int Ultrason Symp, IUS.

[64] Aydin O., Chandran P., Lorsung R.R., Cohen G., Burks S.R., Frank J.A. (2019). The Proteomic Effects of Pulsed Focused Ultrasound on Tumor Microenvironments of Murine Melanoma and Breast Cancer Models. Ultrasound Med Biol.

[65] Zhao X., Wright A., Goertz D.E. (2023). An optical and acoustic investigation of microbubble cavitation in small channels under therapeutic ultrasound conditions. Ultrason Sonochem.

[66] Zhao X., Pellow C., Goertz D.E. (2023). Intravital imaging and cavitation monitoring of antivascular ultrasound in tumor microvasculature. Theranostics.

[67] Frutos Díaz-Alejo J., Gonzalez Gomez I., Earl J. (2022). Ultrasounds in cancer therapy: A summary of their use and unexplored potential. Oncol Rev.

[68] Lomax M.E., Folkes L.K., O’Neill P. (2013). Biological consequences of radiation-induced DNA damage: Relevance to radiotherapy. Clin Oncol.

[69] Santivasi W.L., Xia F. (2014). Ionizing Radiation-Induced DNA Damage, Response, and Repair. Antioxid Redox Signal.

[70] Sørensen B.S., Horsman M.R. (2020). Tumor Hypoxia: Impact on Radiation Therapy and Molecular Pathways. Front Oncol.

[71] Telarovic I., Wenger R.H., Pruschy M. (2021). Interfering with Tumor Hypoxia for Radiotherapy Optimization. J Exp Clin Cancer Res.

[72] Tempany C.M.C., McDannold N.J., Hynynen K., Jolesz F.A. (2011). Focused ultrasound surgery in oncology: Overview and principles. Radiology.

[73] Al-Bataineh O., Jenne J., Huber P. (2012). Clinical and future applications of high intensity focused ultrasound in cancer. Cancer Treat Rev.

[74] Malietzis G., Monzon L., Hand J., Wasan H., Leen E., Abel M. (2013). High-intensity focused ultrasound: advances in technology and experimental trials support enhanced utility of focused ultrasound surgery in oncology. Br J Radiol.

[75] Lindner J.R. (2004). Microbubbles in medical imaging: Current applications and future directions. Nat Rev Drug Discov.

[76] Kiessling F., Fokong S., Koczera P., Lederle W., Lammers T. (2012). Ultrasound microbubbles for molecular diagnosis, therapy, and theranostics. J Nucl Med.

[77] Kiessling F., Bzyl J., Fokong S., Siepmann M., Schmitz G., Palmowski M. (2012). Targeted Ultrasound Imaging of Cancer: An Emerging Technology on its Way to Clinics. Curr Pharm Des.

[78] Kim Y.S., Rhim H., Min J.C., Hyo K.L., Choi D. (2008). High-intensity focused ultrasound therapy: An overview for radiologists. Korean J Radiol.

[79] Sharma D., Czarnota G.J. (2019). Role of acid sphingomyelinase-induced ceramide generation in response to radiation. Oncotarget.

[80] McNabb E., Al-Mahrouki A., Law N., McKay S., Tarapacki C., Hussein F. (2020). Ultrasound-stimulated microbubble radiation enhancement of tumors: Single-dose and fractionated treatment evaluation. Lebedeva I V., editor. PLoS One.

[81] Leong KX. (2022).

[82] Hysi E., Fadhel M.N., Wang Y., Sebastian J.A., Giles A., Czarnota G.J. (2020). Photoacoustic imaging biomarkers for monitoring biophysical changes during nanobubble-mediated radiation treatment. Photoacoustics.

[83] McNabb E., Sharma D., Sannachi L., Giles A., Yang W., Czarnota G.J. (2023). MR-guided ultrasound-stimulated microbubble therapy enhances radiation-induced tumor response. Sci Rep.

[84] Dasgupta A., Saifuddin M., McNabb E., Ho L., Lin L.u., Vesprini D., Karam I., Soliman H., Chow E., Gandhi S., Trudeau M., Tran W., Curpen B., Stanisz G., Sahgal A., Michael Kolios GJC (2023). Sci Rep.

[85] Al-Mahrouki A., Iradji S., Tran W.T., Czarnota G.J. (2014). Cellular characterization of ultrasound-stimulated microbubble radiation enhancement in a prostate cancer xenograft model. DMM Dis Model Mech.

[86] Al-Mahrouki A., Giles A., Hashim A., Kim H.C., El-Falou A., Rowe-Magnus D. (2017). Microbubble-based enhancement of radiation effect: Role of cell membrane ceramide metabolism. Ulasov I, editor. PLoS One.

[87] Shi J., Fu C., Su X., Feng S., Wang S. (2021). Ultrasound-Stimulated Microbubbles Inhibit Aggressive Phenotypes and Promotes Radiosensitivity of esophageal squamous cell carcinoma. Bioengineered.

[88] Ba S., Yu M. (2022). Ultrasound-stimulated microbubbles enhances radiosensitivity of ovarian cancer. Acta radiol.

[89] Deng H., Cai Y., Feng Q., Wang X., Tian W., Qiu S. (2018). Ultrasound-Stimulated Microbubbles Enhance Radiosensitization of Nasopharyngeal Carcinoma. Cell Physiol Biochem.

[90] Kim H.C., Al-Mahrouki A., Gorjizadeh A., Sadeghi-Naini A., Karshafian R., Czarnota G.J. (2014). Quantitative Ultrasound Characterization of Tumor Cell Death: Ultrasound-Stimulated Microbubbles for Radiation Enhancement. Chen X, editor. PLoS One.

[91] El Kaffas A., Nofiele J., Giles A., Cho S., Liu S.K., Czarnota G.J. (2014). Dll4-Notch Signalling Blockade Synergizes Combined Ultrasound-Stimulated Microbubble and Radiation Therapy in Human Colon Cancer Xenografts. Ramchandran R, editor. PLoS One.

[92] Fuks Z., Kolesnick R. (2005). Engaging the vascular component of the tumor response. Cancer Cell.

[93] Sharma D., Czarnota G.J. (2022). Involvement of Ceramide Signalling in Radiation-Induced Tumour Vascular Effects and Vascular-Targeted Therapy. Int J Mol Sci.

[94] Lai P., Tarapacki C., Tran W.T., El K.A., Hupple C., Iradji S. (2016). Breast tumor response to ultrasound mediated excitation of microbubbles and radiation therapy in vivo. Oncosciencencoscience.

[95] Cavalli R., Bisazza A., Trotta M., Argenziano M., Lembo D., Civra A. (2012). New chitosan nanobubbles for ultrasound-mediated gene delivery: preparation and in vitro characterization. Int J Nanomedicine.

[96] Cavalli R., Soster M., Argenziano M. (2016). Nanobubbles: a promising efficient tool for therapeutic delivery. Ther Deliv.

[97] Bhandari P., Novikova G., Goergen C.J., Irudayaraj J. (2018). Ultrasound beam steering of oxygen nanobubbles for enhanced bladder cancer therapy. Sci Rep.

[98] Batchelor D.V.B., Abou-Saleh R.H., Coletta P.L., McLaughlan J.R., Peyman S.A., Evans S.D. (2020). Nested Nanobubbles for Ultrasound-Triggered Drug Release. ACS Appl Mater Interfaces.

[99] Su C., Ren X.J., Nie F., Li T., Lv W., Li H. (2021). Current advances in ultrasound-combined nanobubbles for cancer-targeted therapy: a review of the current status and future perspectives. RSC Adv.

[100] Nittayacharn P., Yuan H.X., Hernandez C., Bielecki P., Zhou H., Exner A.A. (2019). Enhancing Tumor Drug Distribution With Ultrasound-Triggered Nanobubbles. J Pharm Sci.

[101] Zhang J., Chen Y., Deng C., Zhang L., Sun Z., Wang J. (2019). The optimized fabrication of a novel nanobubble for tumor imaging. Front Pharmacol.

[102] Xu J., Salari A., Wang Y., He X., Kerr L., Darbandi A. (2021). Microfluidic Generation of Monodisperse Nanobubbles by Selective Gas Dissolution. Small.

[103] Jafari Sojahrood A., De Leon A.C., Lee R., Cooley M., Abenojar E.C., Kolios M.C. (2021). Toward Precisely Controllable Acoustic Response of Shell-Stabilized Nanobubbles: High Yield and Narrow Dispersity. ACS Nano.

[104] Moore M.J., Bodera F., Hernandez C., Shirazi N., Abenojar E., Exner A.A. (2020). The dance of the nanobubbles: Detecting acoustic backscatter from sub-micron bubbles using ultra-high frequency acoustic microscopy. Nanoscale.

[105] Abenojar E.C., Bederman I., de Leon A.C., Zhu J., Hadley J., Kolios M.C. (2020). Theoretical and Experimental Gas Volume Quantification of Micro- and Nanobubble Ultrasound Contrast Agents. Pharmaceutics.

[106] De Leon A., Perera R., Hernandez C., Cooley M., Jung O., Jeganathan S. (2019). Contrast enhanced ultrasound imaging by nature-inspired ultrastable echogenic nanobubbles. Nanoscale.

[107] Hernandez C., Abenojar E.C., Hadley J., De Leon A.C., Coyne R., Perera R. (2019). Sink or float? Characterization of shell-stabilized bulk nanobubbles using a resonant mass measurement technique. Nanoscale.

[108] Dimcevski G., Kotopoulis S., Bjånes T., Hoem D., Schjøt J., Gjertsen B.T. (2016). A human clinical trial using ultrasound and microbubbles to enhance gemcitabine treatment of inoperable pancreatic cancer. J Control Release.

[109] Wang Y., Li Y., Yan K., Shen L., Yang W., Gong J. (2018). Clinical study of ultrasound and microbubbles for enhancing chemotherapeutic sensitivity of malignant tumors in digestive system. Chinese J Cancer Res.

[110] Lindau D., Gielen P., Kroesen M., Wesseling P., Adema G.J. (2013). The immunosuppressive tumour network: Myeloid-derived suppressor cells, regulatory T cells and natural killer T cells. Immunology.

[111] Tan S., Li D., Zhu X. (2020). Cancer immunotherapy: Pros, cons and beyond. Biomed Pharmacother.

[112] Hayes C. (2021). Cellular immunotherapies for cancer. Ir J Med Sci.

[113] Rosenberg S.A. (2011). Cell transfer immunotherapy for metastatic solid cancer-what clinicians need to know. Nature Reviews. Clin Oncol.

[114] Fan Y., Moon J.J. (2015). Nanoparticle drug delivery systems designed to improve cancer vaccines and immunotherapy. Vaccines.

[115] Kimiz-Gebologlu I., Gulce-Iz S., Biray-Avci C. (2018). Monoclonal antibodies in cancer immunotherapy. Mol Biol Rep.

[116] Trujillo J.A., Sweis R.F., Bao R., Luke J.J. (2018). T cell–inflamed versus Non-T cell–inflamed tumors: a conceptual framework for cancer immunotherapy drug development and combination therapy selection. Cancer Immunol Res.

[117] Duffy M.J., Crown J. (2019). Biomarkers for predicting response to immunotherapy with immune checkpoint inhibitors in cancer patients. Clin Chem.

[118] Lorenzo-Herrero S., López-Soto A., Sordo-Bahamonde C., Gonzalez-Rodriguez A., Vitale M., Gonzalez S. (2018). NK Cell-Based Immunotherapy in Cancer Metastasis. Cancers (Basel).

[119] Marhelava K., Pilch Z., Bajor M., Graczyk-Jarzynka A., Zagozdzon R. (2019). Targeting Negative and Positive Immune Checkpoints with Monoclonal Antibodies in Therapy of Cancer. Cancers (Basel).

[120] Yamamoto T.N., Kishton R.J., Restifo N.P. (2019). Developing neoantigen-targeted T cell–based treatments for solid tumors. Nat Med.

[121] Zhang B.L., Li D., Gong Y.L., Huang Y., Qin D.Y., Jiang L. (2019). Preclinical Evaluation of Chimeric Antigen Receptor-Modified T Cells Specific to Epithelial Cell Adhesion Molecule for Treating Colorectal Cancer. Hum Gene Ther.

[122] Costa R.L.B., Czerniecki B.J. (2020). Clinical development of immunotherapies for HER2+ breast cancer: a review of HER2-directed monoclonal antibodies and beyond. npj. Breast Cancer.

[123] Moufarrij S., Srivastava A., Gomez S., Hadley M., Palmer E., Austin P.T. (2020). Combining DNMT and HDAC6 inhibitors increases anti-tumor immune signaling and decreases tumor burden in ovarian cancer. Sci Rep.

[124] Robert C. (2020). A decade of immune-checkpoint inhibitors in cancer therapy. Nat Commun.

[125] Raskov H., Orhan A., Christensen J.P., Gögenur I. (2021). Cytotoxic CD8+ T cells in cancer and cancer immunotherapy. Br J Cancer.

[126] Gaynor N., Crown J., Collins D.M. (2022). Immune checkpoint inhibitors: Key trials and an emerging role in breast cancer. Sem Cancer Biol.

[127] Escoffre J.-M., Deckers R., Bos C., Moonen C. (2016). Bubble-Assisted Ultrasound: Application in Immunotherapy and Vaccination. Adv Exper Med Biol.

[128] Dobosz P., Stępień M., Golke A., Dzieciątkowski T. (2022). Challenges of the Immunotherapy: Perspectives and Limitations of the Immune Checkpoint Inhibitor Treatment. Int J Mol Sci.

[129] Taefehshokr S., Parhizkar A., Hayati S., Mousapour M., Mahmoudpour A., Eleid L. (2022). Cancer immunotherapy: Challenges and limitations. Pathol - Res Pract.

[130] Unga J., Hashida M. (2014). Ultrasound induced cancer immunotherapy. Adv Drug Deliv Rev.

[131] Mauri G., Nicosia L., Xu Z., Di Pietro S., Monfardini L., Bonomo G. (2018). Focused ultrasound: tumour ablation and its potential to enhance immunological therapy to cancer. Br J Radiol.

[132] Sheybani N.D., Price R.J. (2019). Perspectives on recent progress in focused ultrasound immunotherapy. Theranostics.

[133] Ho Y.J., Li J.P., Fan C.H., Liu H.L., Yeh C.K. (2020). Ultrasound in tumor immunotherapy: Current status and future developments. J Control Release.

[134] Curley C.T., Sheybani N.D., Bullock T.N., Price R.J. (2017). Focused ultrasound immunotherapy for central nervous system pathologies: challenges and opportunities. Theranostics.

[135] Mukhopadhyay D., Ahmed A., Sano C., Awad N., Al Sawaftah N., Husseini G.A. (2021). Ultrasound-Triggered Immunotherapy for Cancer Treatment: An Update. Curr Protein Pept Sci.

[136] Yuan J., Ye D., Chen S., Chen H. (2021). Therapeutic Ultrasound-Enhanced Immune Checkpoint Inhibitor Therapy [Internet]. Front Phys.

[137] Sun S., Tang Q., Sun L., Zhang J., Zhang L., Xu M. (2022). Ultrasound-mediated immune regulation in tumor immunotherapy. Mater Today Adv.

[138] Wu F., Wang Z.B., Lu P., Xu Z.L., Chen W.Z., Zhu H. (2004). Activated anti-tumor immunity in cancer patients after high intensity focused ultrasound ablation. Ultrasound Med Biol.

[139] Hu Z., Yang X.Y., Liu Y., Sankin G.N., Pua E.C., Morse M.A. (2007). Investigation of HIFU-induced anti-tumor immunity in a murine tumor model. J Transl Med.

[140] Wu F., Wang Z.B., De C.Y., Zhou Q., Zhang Y., Xu Z.L. (2007). Expression of tumor antigens and heat-shock protein 70 in breast cancer cells after high-intensity focused ultrasound ablation. Ann Surg Oncol.

[141] Wu F., Zhou L., Chen W.R. (2007). Host antitumor immune responses to HIFU ablation. Int J Hyperth.

[142] Lu P., Zhu X.Q., Xu Z.L., Zhou Q., Zhang J., Wu F. (2009). Increased infiltration of activated tumor-infiltrating lymphocytes after high intensity focused ultrasound ablation of human breast cancer. Surgery.

[143] Zhang Y., Deng J., Feng J., Wu F. (2010). Enhancement of antitumor vaccine in ablated hepatocellular carcinoma by high-intensity focused ultrasound. World J Gastroenterol.

[144] Liu F., Hu Z., Qiu L., Hui C., Li C., Zhong P. (2010). Boosting high-intensity focused ultrasound-induced anti-tumor immunity using a sparse-scan strategy that can more effectively promote dendritic cell maturation. J Transl Med.

[145] Haen S.P., Pereira P.L., Salih H.R., Rammensee H.G., Gouttefangeas C. (2011). More than just tumor destruction: Immunomodulation by thermal ablation of cancer [Internet]. Clin Develop Immunol.

[146] Xia J.Z., Xie F.L., Ran L.F., Xie X.P., Fan Y.M., Wu F. (2012). High-Intensity Focused Ultrasound Tumor Ablation Activates Autologous Tumor-Specific Cytotoxic T Lymphocytes. Ultrasound Med Biol.

[147] Wu F. (2013). High intensity focused ultrasound ablation and antitumor immune response. J Acoust Soc Am.

[148] van den Bijgaart R.J.E., Eikelenboom D.C., Hoogenboom M., Fütterer J.J., den Brok M.H., Adema G.J. (2017). Thermal and mechanical high-intensity focused ultrasound: perspectives on tumor ablation, immune effects and combination strategies. Cancer Immunol Immunother.

[149] Shi G., Zhong M., Ye F., Zhang X. (2019). Low-frequency HIFU induced cancer immunotherapy: tempting challenges and potential opportunities. Cancer Biol Med.

[150] Eranki A., Srinivasan P., Ries M., Kim A.R., Lazarski C.A., Rossi C.T. (2020). High-intensity focused ultrasound (hIFU) triggers immune sensitization of refractory murine neuroblastoma to checkpoint inhibitor therapy. Clin Cancer Res.

[151] Fite B.Z., Wang J., Kare A.J., Ilovitsh A., Chavez M., Ilovitsh T. (2021). Immune modulation resulting from MR-guided high intensity focused ultrasound in a model of murine breast cancer. Sci Rep.

[152] Li X., Khorsandi S., Wang Y., Santelli J., Huntoon K., Nguyen N. (2022). Cancer immunotherapy based on image-guided STING activation by nucleotide nanocomplex-decorated ultrasound microbubbles. Nat Nanotechnol.

[153] Liu S., Zhang Y., Liu Y., Wang W., Gao S., Yuan W. (2023). Ultrasound-targeted microbubble destruction remodels tumour microenvironment to improve immunotherapeutic effect. Br J Cancer.

[154] Endo-Takahashi Y., Negishi Y. (2020). Microbubbles and nanobubbles with ultrasound for systemic gene delivery. Pharmaceutics..

[155] Jiang M., Chen P., Wang L., Li W., Chen B., Liu Y. (2020). cGAS-STING, an important pathway in cancer immunotherapy. J Hematol Oncol.

[156] Singh M.P., Sethuraman S.N., Ritchey J., Fiering S., Guha C., Malayer J. (2019). In-situ vaccination using focused ultrasound heating and anti-CD-40 agonistic antibody enhances T-cell mediated local and abscopal effects in murine melanoma. Int J Hyperth.

[157] Qu S., Worlikar T., Felsted A.E., Ganguly A., Beems M.V., Hubbard R. (2020). Non-thermal histotripsy tumor ablation promotes abscopal immune responses that enhance cancer immunotherapy. J Immunother Cancer.

[158] Hu J., He J., Wang Y., Zhao Y., Fang K., Dong Y. (2022). Ultrasound combined with nanobubbles promotes systemic anticancer immunity and augments anti-PD1 efficacy. J Immunother Cancer.

[159] Schadendorf D., Hodi F.S., Robert C., Weber J.S., Margolin K., Hamid O. (2015). Pooled analysis of long-term survival data from phase II and phase III trials of ipilimumab in unresectable or metastatic melanoma. J Clin Oncol.

[160] Maio M., Grob J.J., Aamdal S., Bondarenko I., Robert C., Thomas L. (2015). Five-year survival rates for treatment-naive patients with advanced melanoma who received ipilimumab plus dacarbazine in a phase III trial. J Clin Oncol.

[161] Yang J.C., Hughes M., Kammula U., Royal R., Sherry R.M., Topalian S.L. (2007). Ipilimumab (anti-CTLA4 antibody) causes regression of metastatic renal cell cancer associated with enteritis and hypophysitis. J Immunother.

[162] Lynch T.J., Bondarenko I., Luft A., Serwatowski P., Barlesi F., Chacko R. (2012). Ipilimumab in combination with paclitaxel and carboplatin as first-line treatment in stage IIIB/IV non-small-cell lung cancer: Results from a randomized, double-blind, multicenter phase II study. J Clin Oncol.

[163] Reck M., Bondarenko I., Luft A., Serwatowski P., Barlesi F., Chacko R. (2013). Ipilimumab in combination with paclitaxel and carboplatin as first-line therapy in extensivedisease-small-cell lungcancer: Results from a randomized, double-blind, multicenter phase 2 trial. Ann Oncol.

[164] Kwon E.D., Drake C.G., Scher H.I., Fizazi K., Bossi A., Van den Eertwegh A.J.M. (2014). Ipilimumab versus placebo after radiotherapy in patients with metastatic castration-resistant prostate cancer that had progressed after docetaxel chemotherapy (CA184-043): A multicentre, randomised, double-blind, phase 3 trial. Lancet Oncol.

[165] Schölch S., Rauber C., Tietz A., Rahbari N.N., Bork U., Schmidt T. (2015). Radiotherapy combined with TLR7/8 activation induces strong immune responses against gastrointestinal tumors. Oncotarget.

[166] Liu Y., Dong Y., Kong L., Shi F., Zhu H., Yu J. (2018). Abscopal effect of radiotherapy combined with immune checkpoint inhibitors. J Hematol Oncol.

[167] Walker R., Poleszczuk J., Pilon-Thomas S., Kim S., Anderson A.A.R.A., Czerniecki B.J. (2018). Immune interconnectivity of anatomically distant tumors as a potential mediator of systemic responses to local therapy. Sci Rep.

[168] Caetano M.S., Younes A.I., Barsoumian H.B., Quigley M., Menon H., Gao C. (2019). Triple therapy with MeRTK and PD1 inhibition plus radiotherapy promotes abscopal antitumor immune responses. Clin Cancer Res.

[169] Chen Y., Gao M., Huang Z., Yu J., Meng X. (2020). SBRT combined with PD-1/PD-L1 inhibitors in NSCLC treatment: a focus on the mechanisms, advances, and future challenges. J Hematol Oncol.

[170] Formenti S.C., Demaria S. (2009). Systemic effects of local radiotherapy. Lancet Oncol.

[171] Carvalho H. de A., Villar R.C. (2018). Radiotherapy and immune response: The systemic effects of a local treatment [Internet]. Clinics.

[172] Finazzi T., Rordorf T., Ikenberg K., Huber G.F., Guckenberger M., Garcia Schueler H.I. (2018). Radiotherapy-induced anti-tumor immune response and immune-related adverse events in a case of recurrent nasopharyngeal carcinoma undergoing anti-PD-1 immunotherapy. BMC Cancer.

[173] Rodríguez-Ruiz M.E., Vanpouille-Box C., Melero I., Formenti S.C., Demaria S. (2018). Immunological Mechanisms Responsible for Radiation-Induced Abscopal Effect. Trends Immunol.

[174] Walle T., Monge R.M., Cerwenka A., Ajona D., Melero I., Lecanda F. (2018). Radiation effects on antitumor immune responses: Current perspectives and challenges [Internet]. Therap Adv Med Oncol.

[175] Balázs K., Badie B., Candéias G. (2019). Radiotherapy-Induced Changes in the Systemic Immune and Inflammation Parameters of Head and Neck Cancer Patients. Cancers (Basel).

[176] Takahashi J., Nagasawa S. (2020). Immunostimulatory effects of radiotherapy for local and systemic control of melanoma: A review. Int J Mol Sci.

